# Polyketide Trimming Shapes Dihydroxynaphthalene‐Melanin and Anthraquinone Pigments

**DOI:** 10.1002/advs.202400184

**Published:** 2024-03-16

**Authors:** Maximilian Schmalhofer, Anna L. Vagstad, Qiuqin Zhou, Helge B. Bode, Michael Groll

**Affiliations:** ^1^ TUM School of Natural Sciences Department of Bioscience Centre for Protein Assemblies Chair of Biochemistry Technical University of Munich 85748 Garching Germany; ^2^ Eidgenössische Technische Hochschule (ETH) Zürich Institute of Microbiology Zürich 8093 Switzerland; ^3^ Department of Natural Products in Organismic Interactions Max Planck Institute for Terrestrial Microbiology 35043 Marburg Germany; ^4^ Molecular Biotechnology Department of Biosciences Goethe University Frankfurt 60438 Frankfurt Germany; ^5^ Department of Chemistry Phillips Universität Marburg 35043 Marburg Germany; ^6^ Center for Synthetic Microbiology (SYNMIKRO) Phillips Universität Marburg 35043 Marburg Germany; ^7^ Senckenberg Gesellschaft für Naturforschung 60325 Frankfurt Germany; ^8^ Present address: Center for Mass Spectrometry and Optical Spectroscopy (CeMOS) Mannheim University of Applied Sciences 68163 Mannheim Germany

**Keywords:** natural products, pigment biosynthesis, polyketide trimming, retro‐Claisen reaction, enzyme catalysis

## Abstract

Pigments such as anthraquinones (AQs) and melanins are antioxidants, protectants, or virulence factors. AQs from the entomopathogenic bacterium *Photorhabdus laumondii* are produced by a modular type II polyketide synthase system. A key enzyme involved in AQ biosynthesis is *Pl*AntI, which catalyzes the hydrolysis of the bicyclic‐intermediate‐loaded acyl carrier protein, polyketide trimming, and assembly of the aromatic AQ scaffold. Here, multiple crystal structures of *Pl*AntI in various conformations and with bound substrate surrogates or inhibitors are reported. Structure‐based mutagenesis and activity assays provide experimental insights into the three sequential reaction steps to yield the natural product AQ‐256. For comparison, a series of ligand‐complex structures of two functionally related hydrolases involved in the biosynthesis of 1,8‐dihydroxynaphthalene‐melanin in pathogenic fungi is determined. These data provide fundamental insights into the mechanism of polyketide trimming that shapes pigments in pro‐ and eukaryotes.

## Graphical abstract

Vital Pigment Functions: Anthraquinones and dihydroxynaphthalene‐melanins play roles in cancer therapy or act as virulence factors. Protein crystallography, along with structure‐based mutagenesis and activity assays, provides atomic insights into polyketide trimming, ring formation, and thioester hydrolysis during AQ and DHN‐melanin biosynthesis in both prokaryotes and eukaryotes.

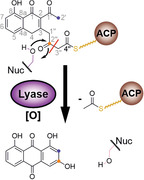

## 1 Introduction

**Figure 1:**
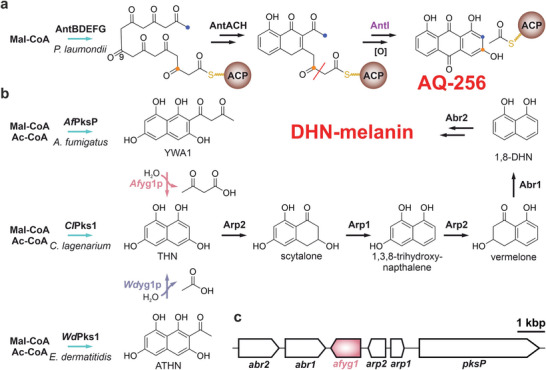
*Af*yg1p, *Wd*yg1p, and *Pl*AntI are involved in the biosynthesis of DHN‐melanin and AQ‐256. a) Proposed reaction sequence catalyzed by *Pl*AntI producing AQ‐256. b) The DHN‐melanin pathway. Type I PKS systems (blue arrows) forming YWA1 (*Af*PksP in *A. fumigatus*), 1,3,6,8‐tetrahydroxy‐naphthalene (THN, *Cl*Pks1 in *C. lagenarium*), or acetyl‐THN (ATHN, *Wd*Pks1 in *E. dermatitidis*) are essential for product formation. Shown are biosynthetic enzymes of *A. fumigatus*. c) Gene cluster from *A. fumigatus* involved in DHN‐melanin formation, including *abr1* (multicopper oxidase), *abr2* (laccase), *ayg1* (hydrolase), *arp2* (hydroxynaphthalene reductase), *arp1* (scytalone dehydratase), and *pksP* (type I PKS system). Mal‐CoA = malonyl‐coenzyme A, Ac–CoA = acetyl‐coenzyme A.

Polyketides represent a large group of secondary metabolites with possible medical, agricultural, and industrial applications and are produced from C_2_ building blocks via polyketide synthases (PKSs). One important group of polyketides is anthraquinones (AQs), which have diverse bioactivities.^[^ ^1^ ^]^ For instance, the scaffold of AQ is part of anthracyclines, a class of diverse anticancer agents.^[^ ^2^ ^]^ A type II PKS system produces the natural product AQ‐256 in the entomopathogenic bacterium *Photorhabdus laumondii* (*Pl*).^[^ ^3^ ^]^ In the first step, the α/β‐heterodimeric ketosynthase *Pl*AntDE assembles a linear octaketide bound to the acyl carrier protein (ACP) *Pl*AntF^holo^ by condensing malonyl‐CoA precursors onto a 4′‐phosphopantetheine prosthetic group (**Figure** **1**a).^[^ ^4^ ^]^ Then, the ketoreductase *Pl*AntA acts at C9, and the aromatases/cyclases (ARO/CYC) *Pl*AntH and *Pl*AntC form and aromatize the ACP‐bound bicycle. The ultimate steps in the biosynthesis of AQ‐256 involve an unusual chain‐length shortening of the octaketide to the final heptaketide and the assembly of the third aromatic ring. This complex reaction sequence consists of i) ACP‐offloading, ii) polyketide trimming, and iii) cyclization, all catalyzed by the α/β‐lyase *Pl*AntI.^[^ ^5^ ^]^

Intriguingly, C─C bond cleavage in polyketide precursor molecules has also been identified in pathogenic fungi during the biosynthesis of 1,8‐dihydroxynaphthalene (DHN)‐melanin (Figure 1b). These natural products are a central component of dark cell‐wall pigments. They are associated with virulence in various hosts, where pigment disruption affects pathogen‐host recognition and fungal pathogenicity, suggesting DHN melanin biosynthesis is a promising target for antifungal drug development.^[^ ^6^ ^]^ A key metabolite of fungal melanin biosynthesis is 1,3,6,8‐tetrahydroxynaphthalene (THN), which undergoes two rounds of enzymatic reduction and dehydration to yield DHN (Figure 1b,c).^[^ ^7^ ^]^ The importance of THN is highlighted by the presence of convergent biosynthetic routes to this common metabolite. For example, in *Aspergillus fumigatus* (*Af*), the type I PKS *Af*PksP produces the naphthopyrone heptaketide YWA1, whose acetoacetyl side chain is removed by the serine hydrolase *Af*yg1p (Figure 1b).^[^ ^7^, ^8^ ^]^ In contrast, in the zoonotic pathogen *Wangiella* (*Exophiala*) *dermatitidis* (*Wd*), the acetyl group of the hexaketide 2‐acetyl‐1,3,6,8‐tetrahydroxynaphthalene (ATHN), *Wd*PKS product, is hydrolyzed by the *Af*yg1p homolog *Wd*yg1p (Figure 1b).^[^ ^9^ ^]^ *Cl*Pks1 in *Colletotrichum lagenarium* (*Cl*) synthesizes THN without additional enzymes, defining yet another fungal pathway to yield this important melanin precursor (Figure 1b).^[^ ^10^ ^]^ Strikingly, the unusual ability of the *Cl*Pks1 thioesterase domain to catalyze tandem reactions by breaking and forming C─C bonds by a single enzymatic domain is reminiscent of the biosynthesis of AQ‐256 via the bacterial type II PKS system (Figure 1a). In each case, the mechanism involves a retro‐Claisen truncation reaction, as well as a Dieckmann condensation, in which a cyclic ACP‐linked intermediate is further processed to the mature natural product (Figure 1a).^[^ ^5^ ^]^

The present study aims to gain mechanistic insights into the underlying binding and catalytic modes in this class of α/β‐lyases/hydrolases. While type I iterative PKSs, such as *Cl*Pks1, are multifunctional enzymes whose catalytic domains collectively coordinate aromatic polyketide assembly, *Pl*AntI, as well as *Af*yg1p and *Wd*yg1p, are discrete enzymes for which the reaction mechanisms are simpler to study. Using a structural and mutagenic approach combined with activity studies, we analyzed here the three‐step reaction of *Pl*AntI in offloading, polyketide trimming, and aromatic ring formation of its ACP‐bound substrate, as well as the catalytic function of the fungal hydrolases *Af*yg1p and *Wd*yg1p. Complex structures of *Pl*AntI, *Af*yg1p, and *Wd*yg1p bound to various substrates, products, surrogates, inhibitors, and compounds mimicking the sp^3^‐hybridized transition state were compared, providing snapshots of catalysis and binding modes for each enzyme and yielding insights into the production of pigments in prokaryotes and eukaryotes.

## 2 Results and Discussion

### 2.1 The Specificity Pocket of *Pl*AntI

**Figure 2:**
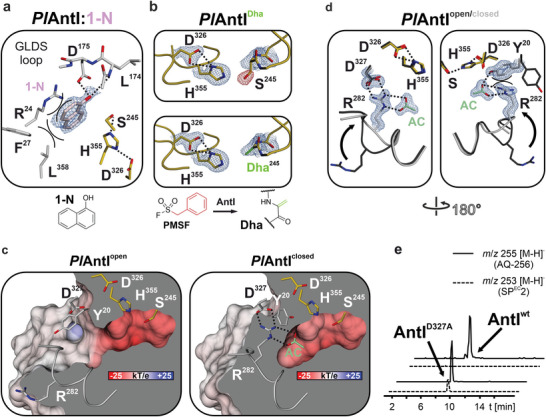
Lyase *Pl*AntI is involved in AQ‐256 biosynthesis. a) Atomic view of *Pl*AntI's active site complexed with 1‐naphthol (*Pl*AntI:1‐N, PDB ID: 8QBH, 2.05 Å, light pink sticks). The 2F_o_‐F_c_ electron density map is shown as a blue mesh and contoured to 1 σ. H─bonds are indicated as black dotted lines and cation‐pi/pi‐stacking interactions are highlighted in black (see Experimental Section for further details). b) Active site of *Pl*AntI (*Pl*AntI^DHA^, PDB ID: 8QD6, 1.7 Å) shows signals in the negative F_o_─F_c_ electron difference map (red mesh, contoured to 3 σ, upper panel) after conversion with SFs. The dehydroalanine (DHA)‐modification (green sticks) is modeled in the 2F_o_─F_c_ electron density map (blue mesh, contoured to 1.8 σ, lower panel). c) *Pl*AntI surface representations of the closed (right panel, *Pl*AntI^closed^, PDB ID: 8QBI, 1.55 Å) and open (left panel, PDB ID: 6HXA) state.^[^ ^5^ ^]^ The gating residues Tyr20, Arg282, and Asp327, as well as the active‐site residues, are presented in sticks (acetate (AC) in green). d) Front (left panel) and back (right panel) view of the gate. The black arrow emphasizes the large movement of Arg282. e) *E. coli* expressing the *ant* biosynthetic gene cluster produces AQ‐256 (black line). The replacement of *Pl*AntI with the gate mutant *Pl*AntI^D327A^ results in shunt products (dashed line). Shown are HPLC/MS analyses with extracted ion chromatograms for AQ‐256 (*m*/*z* 255 [M‐H]^−^) and the shunt product aloesaponarin II (SP^EC^2, *m*/*z* 253 [M‐H]^−^ see also Figure S2, Supporting Information).

The previously described structural analysis of *Pl*AntI provided the first molecular insights into this exceptional class of polyketide‐trimming enzymes.^[^ ^5^ ^]^ The reaction catalyzed by this lyase is crucial for forming the tricyclic aromatic ring in anthraquinone biosynthesis (AQ‐256), but the proposed mechanism is based solely on theoretical calculations and needs experimental validation. Since the natural substrate of *Pl*AntI – a highly reactive, ACP‐bound modified octaketide (Figure 1a) is not accessible, we focused our study on a stable surrogate. We determined the X‐ray structure of *Pl*AntI in complex with the hydroxynaphthalene ligand 1‐naphthol (*Pl*AntI:1‐N, PDB ID: 8QBH, 2.05 Å resolution, **Figure** **2**a; Table S9, Supporting Information). Upon ligand binding, no major structural rearrangements are observed in *Pl*AntI, and the catalytic triad remains perfectly aligned (rmsd = 0.4 Å over 374 C^α^‐atoms). The complex structure displays an open conformation (*Pl*AntI^open^), and 1‐N is buried in a cavity at the interface between the N‐─ and C‐domains (Figure S1, Supporting Information)). The planar naphthalene ring is cation‐pi‐stacked with Arg24 (distance 3.5 Å), while the hydroxyl group of the ligand is in proximity to the catalytic nucleophile (Ser245OH^γ^, 3.4 Å) and interacts with the GLDS loop motif of Gly173‐Ser176. Thus, the specificity pocket in *Pl*AntI consists of the GLDS β‐turn, with Asp175OH^δ^ (3.0 Å) and Leu174NH (3.5 Å) forming interactions with 1‐OH (Figure 2a).

### 2.2 Conversion of the *Pl*AntI Active‐Site Nucleophile into Dehydroalanine (DHA)

Next, the catalytic center of *Pl*AntI was probed with the electrophilic sulfonyl fluorides (SFs) phenylmethane‐SF (PMSF) and phenyl‐SF (PSF). We expected a covalent adduct to form at Ser245 through an attack of this active‐site nucleophile on the sulfur atom of the inhibitor with the release of fluoride. However, structural analyses with both SF‐ligands revealed empty substrate‐binding channels, and the F_o_─F_c_ map displayed strong negative electron density at the side chain of Ser245 (Figure 2b, upper panel). These results suggest a chemical modification of the catalytic center in *Pl*AntI that is analogous to the addition‐elimination reaction for PMSF‐induced conversion of Ser195 in the serine protease thrombin to dehydroalanine (DHA).^[^ ^11^ ^]^ Thus, treatment of *Pl*AntI with PMSF (*) or PSF (^#^) appears to also introduce a DHA residue into the lyase via a transiently sulfonylated active‐site serine (*Pl*AntI^DHA,^*, PDB ID: 8QD6,1.7 Å resolution and *Pl*AntI^DHA,#^, PDB ID: 8QD5, 1.8 Å resolution, Figure 2b, lower panel, and Table S9 (Supporting Information); DHA‐formation was confirmed by mass spectrometry Figure S6 (Supporting Information).

### 2.3 *Pl*AntI Adopts Different Conformations During Catalysis

Intriguingly, the *Pl*AntI^DHA^ structure reveals rearrangements near the catalytic site. Therefore, we screened data sets obtained during our trials for evidence of alternative protein conformations. Indeed, we could identify a fully closed state (*Pl*AntI^closed^, PDB ID: 8QBI, 1.55 Å resolution, Figure 2c, left panel, Figure S1 and Table S9, Supporting Information), in which Arg282 flips 90° inward and Tyr20 rotates by 120° to separate the active site from the bulk solvent. As a result, Arg282 forms strong salt bridges and cation‐pi‐interactions with the gating residues Asp327 and Tyr20, respectively (Figure 2c, left panel, and Figure 2d). Notably, the gating residue Arg282 is further stabilized by an acetate group in the closed‐state structure (see below), whereas in *Pl*AntI^open^ (Figure 2c, right panel and Figure 2d, dark grey), Arg282 and Tyr20 point outward to facilitate substrate access or product release. Intrigued by these two conformations, we wanted to investigate the importance of the gating residues for AQ production. Previous studies have described that AQ‐256 can be generated by heterologous production of the biosynthetic gene cluster (BGC) *antABCDEFGHI* in *E. coli*.^[^ ^5^, ^12^ ^]^ When wild‐type *antI* from the modified *E. coli* strain was replaced by Ser245Ala or His355Ala catalytic‐residue mutants, heptaketide AQ‐256 was absent, and the octaketide shunt product aloesaponarin II (SP^EC^2) was formed (Figure S2, Supporting Information).^[^ ^5^ ^]^ Strikingly, the suspected catalytic Asp326Ala mutant had a similar product profile to the wild‐type production strain, which suggests that Ser245‐His355 functions as a catalytic diad. In this study, the product output of an *antI* ^D327A^ gating variant mutant strain was compared to the wild‐type and generated ≈30% octaketide shunt species in addition to the authentic AQ‐256 product (Figure 2e), highlighting the importance of the gating mechanism during catalysis by *Pl*AntI.

Taken together, our mutational and structural findings from the 1‐N‐bound *Pl*AntI^open^, the SF‐mediated conversion of *Pl*AntI to *Pl*AntI^DHA^, and *Pl*AntI^closed^ provide insights into sophisticated cooperativity of AntI catalysis that involves transitions between open and closed enzyme states crucial to coordinating i) trimming of the intermediate to a heptaketide, ii) offloading of the bicyclic ketide from the acyl carrier protein *Pl*AntF, iii) formation of the third aromatic anthraquinone ring, and iv) product release of AQ‐256. The open form of *PI*AntI is implicated in substrate binding, polyketide trimming, and product release, while the closed form likely facilitates the Dieckmann condensation to the anthraquinone.

In contrast to the sequential reaction sequence required for *Pl*AntI catalysis, the hydrolases *Af*yg1p and *Wd*yg1p from *A. fumigatus* and *E. dermatitidis* (Figures 1b and 2a) act on diffusible polyketide substrates to directly catalyze the shortening of polyketide building blocks. Since these fungal proteins convert their substrate in the absence of an ACP and lack an ongoing cyclization reaction, mechanistic insights into the C─C bond cleavage are more accessible.

### 2.4 The Crystal Structures of the Hydrolases *Af*yg1p and *Wd*yg1p

**Figure 3:**
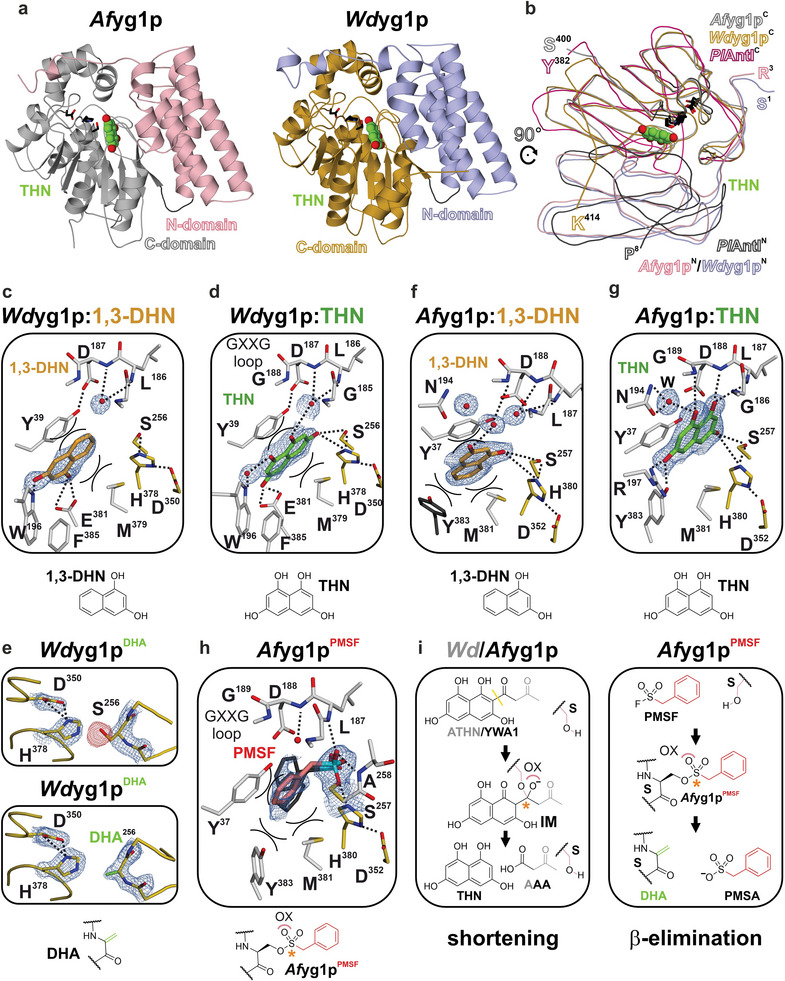
Lyases *Af*yg1p and *Wd*yg1p involved in THN biosynthesis are accessible and active in crystalline form. a) Ribbon drawings of *Af*yg1p (left panel, PDB ID: 8QD1, 1.7 Å) and *Wd*yg1p (right panel, PDB ID: 8QD7, 1.85 Å). The α‐helix bundle (N‐domain, residues 3–140 (*Af*yg1p) and residues 1–139 (Wdyg1p)) are in pink and blue, while the C‐terminal α/β‐hydrolase (C‐domain, residues 145–394 (*Af*yg1p) and residues 144–393 (*Wd*yg1p)) are shown in gray and yellow, respectively. THN is the reaction product of *Af*yg1p and *Wd*yg1p (highlighted as a balls‐and‐sticks model, carbon atoms in green). b) Ribbon plot of *Af*yg1p (residues 3–400) and *Wd*yg1p (residues 1–393) superimposed with *Pl*AntI (residues 7–381, depicted in black and red, PDB ID: 6HXA).^[^ ^5^ ^]^ Atomic view of either 1,3‐DHN (carbon atoms in orange) or THN in the specificity pockets of *Wd*yg1p (left) and *Af*yg1p (right). Ligands do not induce structural rearrangements (rmsd = 0.2 Å over 375–410 C^α^‐atoms). c) Complex structure of *Wd*yg1p:1,3‐DHN (PDB ID: 8QD8, 1.65 Å) d) *Wd*yg1p:THN, PDB ID: 8QD9, 1.95 Å). e) Upper panel: The active site of *Wd*yg1p (*Wd*yg1p^DHA^, PDB ID: 8QDA, 1.85 Å) depicts strong peaks in the F_o_─F_c_ electron difference map (red mesh, contoured to 3 σ) after conversion with SFs. Lower panel: The dehydroalanine (DHA)‐modification (green sticks) is modeled in the 2F_o_─F_c_ electron density map (blue mesh, contoured to 1.8 σ). f) *Af*yg1p:1,3‐DHN, PDB ID: 8QD2, 1.75 Å and g) *Af*yg1p:THN, PDB ID: 8QD3, 1.7 Å. h) Atomic view of modified active‐site serine in *Af*yg1p^PMSF^ (Chain B, PDB ID: 8QD4, 1.8 Å). The benzyl group and phosphate atoms are depicted as red and cyan sticks, respectively. Different orientations of the benzyl moiety in four monomers of the AU are shown in gray. i) Scheme of the putative intermediate bound to *Af*yg1p (left panel). The sp^3^‐hybridized atom is highlighted by an asterisk (orange); O^−^ is stabilized in the oxyanion hole (OX, pink curve). PMSF forms a sulfonate ester (asterisk) with the active‐site serine, which results in DHA (right panel, β‐elimination).

Homodimeric *Af*yg1p and *Wd*yg1p were successfully crystallized and used to collect native datasets to 1.7 Å (PDB ID: 8QD1) and 1.85 Å (PDB ID: 8QD7) resolutions, respectively (**Figure** **3**; Figure S3 and Table S10, Supporting Information). Single‐wavelength anomalous diffraction (SAD) was performed to obtain experimental phases for *Af*yg1p, which was used as a model for Patterson search calculations of the *Wd*yg1p structure. Both enzymes consist of an N‐domain and a C‐domain connected with a short linker (Figure 3a). The N‐domains exhibit an α‐helix bundle, whereas the C‐domains adopt a classical α/β‐hydrolase topology. The amino acid sequences of *Af*yg1p and *Wd*yg1p have 40% sequence identity, which explains the similarity in their overall folds and active‐site residues (rmsd = 1.1 Å over 382 C^α^‐atoms, Figure 3b; Figure S4, Supporting Information). Although both enzymes differ significantly from *Pl*AntI in terms of catalysis and primary sequence, their superposition shows a perfect match of secondary structure elements (rmsd = 1.6 Å over 244 C^α^‐atoms for *Af*yg1p and rmsd = 1.8 Å over 247 C^α^‐atoms for *Wd*yg1p, Figure 3b and Figure S4, Supporting Information). In summary, the N‐ and C‐domains in all three enzymes are oriented in a back‐to‐back fashion forming an interface that encloses the intramolecular binding cavity next to the active site (Figure 3b). The catalytic triad is formed exclusively by the α/β‐hydrolase domain with the nucleophilic serine located in the consensus GX**S**XG esterase motif. The resulting Ser‐His‐Asp network is perfectly aligned with distances in the range of 2.5–2.8 Å between the active‐site residues.

### 2.5 The Specificity Pocket and Active Site of *Wd*yg1p

To investigate the specificity pocket and the underlying binding modes of *Wd*yg1p, conjugated naphthalenes substituted with two hydroxyl‐groups and the product 1,3,6,8‐tetrahydroxynaphthalene (THN) were screened as potential ligands. While co‐crystallization experiments with 1,6‐, 1,8‐, and 2,7‐DHN only display the apo state, the X‐ray structure of *Wd*yg1p in complex with 1,3‐DHN was determined to 1.65 Å resolution (*Wd*yg1p:1,3‐DHN, PDB ID: 8QD8, Figure 3c and Table S11, Supporting Information). The ligand is buried at the interface of the N‐ and C‐domain (Figure 3b). The 1─OH of 1,3‐DHN is H‐bonded to Glu381O^ε^ (2.3 and 3.5 Å), and the 3─OH interacts with the indole nitrogen of Trp196 (3.1 Å), whereas the planar naphthalene ring system is pi‐stacked between Met379 and Tyr39. Next, we solved the *Wd*yg1p crystal structure in complex with its product, THN (*Wd*yg1p:THN, PDB ID: 8QD9, 1.95 Å resolution, Figure 3d; Table S11, Supporting Information). Here, 3─OH of the ligand is H─bonded with the active‐site Ser256OH^γ^ (3.2 Å) and His378N^ε^ (3.2 Å), while 1─OH coordinates via a water molecule to Gly185NH. In addition, 6─OH of THN interacts with Glu381O^ε^ (2.3 and 2.5 Å), and its 8─OH coordinates with the indole nitrogen atom of Trp196 (via a water molecule, Figure 3d).

Taken together, THN and 1,3‐DHN are located at the edge of *Wd*yg1p's specificity pocket and facilitate different in‐plane orientations. Thereby, the bottom of the specificity pocket is defined by Glu381 and Trp196, which spans 7 × 12 × 12 Å^3^. Like in *Pl*AntI, the treatment of *Wd*yg1p with PMSF (*) or PSF (^#^) leads to the β‐elimination mediated conversion of the active‐site Ser256 to DHA (*Wd*yg1p^DHA,^*, PDB ID: 8QDA, 1.85 Å and *Wd*yg1p^DHA,#^, PDB ID: 8QDB, 1.85 Å, Figure 3e,i; Table S11, Supporting Information).

### 2.6 The Specificity Pocket of *Af*yg1p

In parallel, we determined the structure of *Af*yg1p:1,3‐DHN to a resolution of 1.75 Å (PDB ID: 8QD2, Figure 3f; Table S12, Supporting Information). The specificity pocket of *Af*yg1p and *Wd*yg1p share many conserved residues and molecule‐binding features with some notable differences. The electron density map displays the pi‐stacking of the planar naphthalene ring systems with Met381 and Tyr37 (analogous to Met379 and Tyr39, respectively, of *Wd*yg1p). Here, the hydroxyl groups of the surrogate are oriented toward the active site of *Af*yg1p but form only loose H─bonding interactions with Ser257O^γ^ and His380N^ε^ (3.7 and 3.9 Å, Figure 3f). Co‐crystallization experiments of the *Af*yg1p^S257A^ mutant with 1,3‐DHN support these minor interactions with the active site since the ligand binds identically without the nucleophilic hydroxyl group of Ser257. In contrast to *Wd*yg1p, the cavity in *Af*yg1p:1,3‐DHN is enclosed by Tyr383, which is rotated by 90° compared to the *apo* state and extends additional pi–pi‐stacking interactions with 1,3‐DHN (3.7 Å, Figure 3f).

Next, the structure of *Af*yg1p in complex with its reaction product THN was analyzed (*Af*yg1p:THN, PDB ID: 8QD3,1.7 Å resolution, Figure 3g; Table S12, Supporting Information) with the ligand fully defined in the 2F_o_─F_c_ electron density map. The naphthalene ring performs only weak pi‐stacking with Met381 and Tyr37. The binding mode shifts toward an extensive network of H─bonding interactions between *Af*yg1p and the four hydroxyl groups of THN (Figure 3g). The interplay formed by the GLDG motif (β‐turn, residues Gly186‐Gly189), the active site, and the amino acids at the specificity pocket are prominent. Here, 1─OH of the ligand coordinates with Gly186NH (3.7 Å), Leu187NH (3.2 Å), Asp188NH (3.3 Å), Gly189NH (3.0 Å), and the carboxylic side chain of Asp188O^δ^ (3.7 Å), while 8─OH is H─bonded to Gly186NH (3.7 Å), Gly189NH (3.7 Å), and the side chain of Asn194 via a water molecule. Furthermore, the 3─OH of THN cooperates with the side chains of Ser257OH^γ^ (3.5 Å), His380N^ε^ (3.6 Å), Asp188O^δ^ (3.6 Å), and Tyr37OH^η^ (3.4 Å). The 6─OH forms tight interactions with Arg197N^η^ (2.9 Å) and Tyr383OH^η^ (2.3 Å), the residue alternatively involved in pi–pi‐stacking with the naphthalene ring of 1,3‐DHN. Strikingly, in comparison with the *Af*yg1p:1,3‐DHN complex structure, THN is rotated 90° in‐plane and shifted almost 4 Å deeper into the pocket (Figure S5a, Supporting Information), resulting in an open and spacious binding cavity between the active site and the surrounding bulk solvent that extends ≈7 × 10 × 12 Å^3^.

Taken together, the specificity pocket of *Af*yg1p is entirely occupied by the product, whereas in *Af*yg1p^apo^, each position matching the hydroxyl groups 1─OH, 6─OH, and 8─OH of THN and 1─OH of 1,3‐DHN (analogous to 3─OH in THN) is filled by a water molecule. Thus, the solvent release would entropically favor substrate binding (Figure S5b, Supporting Information).

### 2.7 SFs Mimic a Tetrahedral Intermediate Inside of *Af*yg1p

Interestingly, treatment with PMSF did not result in the elimination of a DHA residue in *Af*yg1p, as had occurred at the catalytic serine for *Pl*AntI and *Wd*yg1p. The F_o_─F_c_ electron density map instead illustrates the inhibitor bound to the nucleophilic Ser257O^γ^ as a sulfonate ester (*Af*yg1p^PMSF^, PDB ID: 8QD4, 1.8 Å resolution, Figure 3h,i; Table S12, Supporting Information). Compared with *Af*yg1p^apo^, no structural rearrangements take place upon PMSF binding (rmsd = 0.2 Å over 397 C^α^‐atoms), and, despite the covalent attachment, the catalytic triad remains perfectly aligned with distances of 2.9 Å (Ser257O^γ^ and His380N^ε^) and 2.5 Å (His380NH^ε^ and Asp352O^δ^). Here, the bound sulfonate ester adopts a tetrahedral orientation that mimics parts of a late reaction state (Figure 3i), with the oxyanion hole of *Af*yg1p formed by the coordinating backbone amides of Leu187 (3.0 Å) and Ala258 (3.0 Å, Figure 3h; Figure S5c, Supporting Information). Moreover, a dipole provided by helix 8 (nucleophilic elbow, residues Ser257‐His270) intensifies the interactions, while pi‐stacking between the benzyl group of PMSF and Tyr37, Met381, and Tyr383 is less pronounced. Consequently, the aryl moiety is mobile and adopts multiple conformations with an 80° rotation in‐plane (Figure 3h).

### 2.8 An Elongated Tunnel of *Af*yg1p Stabilizes the Acetoacetyl Ketides

**Figure 4:**
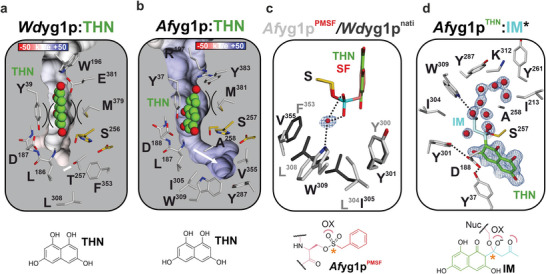
The naphthalene and (aceto)acetyl specificity pockets of *Af*yg1p and *Wd*yg1p. Sliced surface representation with electrostatic potentials of THN (carbon atoms in green) bound to a) *Wd*yg1p and b) *Af*yg1p (pi‐stacking is shown in semicircles, a white arrow emphasizes the acetoacetyl‐tunnel). c) Superposition of *Wd*yg1p^apo^ (gray sticks) and *Af*yg1p^PMSF^ (white sticks, red spheres depict water molecules). d) Schematic view of the acetoacetyl binding site illustrating the *Af*yg1p:THN structure with the modeled tetrahedral intermediate (IM^*^, blue and green sticks).

Following the identification of the naphthalene specificity pocket, the binding sites for the acetyl and (aceto)acetyl appendages subject to hydrolysis were analyzed for the fungal hydrolases (**Figure** **4**). *Wd*yg1p and *Af*yg1p cleave the hexaketide acetyl‐THN (ATHN) and the naphthopyrone heptaketide YWA1, respectively, to the pentaketide THN. Therefore, the substrate's acetyl or acetoacetyl group must be accommodated next to the active site. Interestingly, both hydrolases provide a hydrophobic tunnel beside the naphthalene specificity pocket (Figure 4a,b). The 400 Å^3^ cavity of *Wd*yg1p is confined by the amino acids Thr257, Leu304, Leu308, and Phe353, which could accommodate an acetyl group (Figure 4a). Consistent with the more extended polar substrate, the acetoacetyl binding site in *Af*yg1p is less hydrophobic, and the chamber formed by Ala258, Tyr287, Ile305, Trp309, and Val355 is increased in size (800 Å^3^, Figure 4b). Thereby, Trp309 is located adjacent to the oxyanion hole and coordinates a well‐defined water molecule that is also present in *Af*yg1p^apo^, *Af*yg1p^PMSF^, and *Af*yg1p:1,3‐DHN, as well as in *Af*yg1p:THN (Figure 4c). Thus, the indole group of Trp309 likely stabilizes the distal carbonyl oxygen atom in the acetoacetyl sidechain. The unique specificity pockets of *Af*yg1p and *Wd*yg1p rationalize their choice of compounds. Despite discrepancies in the substrate binding pockets, the numerous snapshots of *Af*yg1p and *Wd*yg1p complex structures suggest a uniform polyketide shortening mechanism in this class of hydrolases (see conclusions below).

## 3 Conclusion

**Figure 5:**
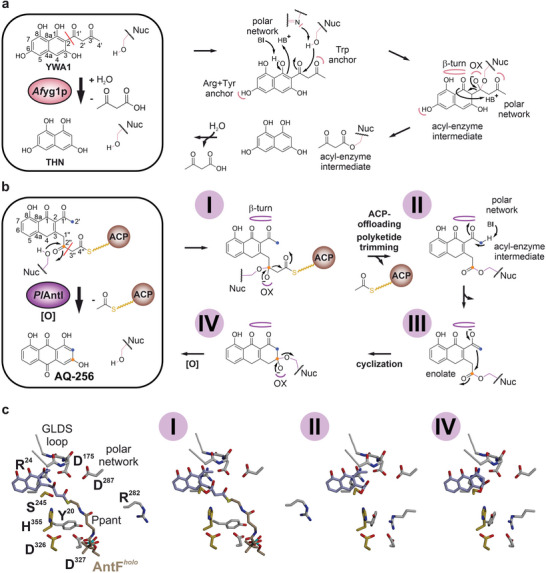
Proposed enzyme mechanism for *Af*yg1p, *Wd*yg1p, and *Pl*AntI. a) In *Af*yg1p and *Wd*yg1p, C─C bond breakage proceeds via a tetrahedral intermediate, Nuc = active‐site nucleophile (Ser), Trp anchor = pocket that stabilizes the aceto moiety of YWA1, hinge = β‐turn that stabilizes the 1‐ and 8‐hydroxyl groups, OX = oxyanion hole, polar network = responsible for keto‐enol tautomerism, B = base, and BH^+^ = protonated base, red line = cleaved C─C bond. The proposed mechanism is extended from Fujii et al.^[^ ^7b^ ^]^ b) The proposed reaction mechanism of *Pl*AntI includes ACP‐offloading, polyketide trimming, and cyclization. The numbering of the C‐atoms is equivalent to the numbering of YWA1. The proposed mechanism is extended from Zhou et al.^[^ ^5^ ^]^ c) Stick representations of *Pl*AntI intermediates (states I, III, and IV). For clarity, only the residues in an unbound state are labeled.

In some fungal DHN‐melanin pigment formation, type I PKS thioesterase domains first catalyze ACP‐offloading and Dieckmann condensation to release 2‐substituted naphthyl intermediates, which are subject to polyketide trimming to THN in separate downstream reactions by *Af*yg1p‐ and *Wd*yg1p‐type α/β‐hydrolases (Figures 1 and **5**). In contrast, *Pl*AntI is the final enzyme in the unusual bacterial type II PKS biosynthesis of AQ‐256, where it coordinates C2″–C3″ bond cleavage, as well as detachment from the discrete ACP *Pl*AntF*^holo^* and transitions to the closed state to facilitate cyclization of the third aromatic ring.

The determined structures of *Af*yg1p highlight several key features: i) the orientation of the conjugated pi‐system in the naphthalene specificity pocket is determined by tight coordination of the four hydroxyl groups (*Af*yg1p:THN); ii) the sp^3^‐hybridized intermediate state of the bound PMSF inhibitor enables the assignment of the oxyanion hole (*Af*yg1p^PMSF^); iii) Trp309 stabilizes the acetoacetyl moiety that is cleaved during catalysis (*Af*yg1p^apo/PMSF^ and *Af*yg1p:1,3‐DHN/THN). With these experimental results in hand, the tetrahedral intermediate was modeled in its keto form (*Af*yg1p:IM*) using the alignment of THN and the water molecule next to Trp309 as additional anchor points (Figure 4d). Based on these atomic insights, we propose the following mechanism (Figure 5a): First, the substrate YWA1 in its side‐chain open form binds to *Af*yg1p via all four hydroxyl groups of the naphthalene ring system. Asp352 and His380 of the active triad deprotonate Ser256OH^γ^. The resulting nucleophile attacks the electrophilic keto group (1′‐carbonyl), and the emerging oxyanion is stabilized by the backbone amides Leu187NH and Ala258NH. In addition, the polar network (Tyr37OH^η^, Asp188O/OH^δ^, and Tyr301OH^η^) drives the conversion of the enol to the ketone group at C1. These residues are in proximity to 1─OH and C2 (3.7 and 3.2 Å to Asp188O/OH^δ^, respectively) and facilitate the deprotonation of 1─OH and the successive protonation at C2. The keto‐form of the intermediate allows the reshuffling of electrons back into the ring system, which is instrumental for the C2─C1' bond cleavage. Last, the protonation of O1 takes place via the polar network to form the conjugated THN ring. Hydrolysis of the Ser256‐bound acetoacetyl group finally restores the catalytic center and releases the products THN and 3‐oxobutanoate. This mechanism is most likely transferable to *Wd*yg1p due to the high similarity of their active‐site architectures and the GLDG binding motifs (Figure 4d; Figure S4, Supporting Information). Notably, the entire reaction in the hydrolases *Af*yg1p and *Wd*yg1p happens in an open state (Figure S1, Supporting Information).

*Pl*AntI's reaction trajectory and substrate significantly differ from the fungal hydrolases *Af*yg1p and *Wd*yg1p. Nevertheless, the similar overall folds and orientation of catalytic residues support that the shortening of the polyketide in *Pl*AntI follows a similar mechanism. Thus, based on the accumulated results, we favor a reaction mechanism where polyketide trimming and ACP release co‐occur first on the ACP‐bound bicyclic intermediate to give acetyl‐AntF^holo^ as a co‐product of the truncated acyl‐enzyme intermediate on Ser245 of AntI. Then transition to the closed state is followed by Dieckmann condensation to the third aromatic ring to give the final AQ‐256 product. In more detail, the activated Ser245O^γ−^ attacks the C2″‐atom of the substrate, and the oxyanion hole (Leu174NH and Phe246NH) stabilizes the formed negative charge. However, rather than pushing electrons toward the ring system, as analogous to *Af*yg1p or *Wd*yg1p catalysis (Figure 5b–I), *Pl*AntI instead breaks the C2″–C3″ bond by the release of acetyl‐AntF*^holo^* in a retro‐Claisen reaction. The remaining C_14_ intermediate remains covalently bound to Ser245 via an ester bond (**II**). In contrast to *Af*yg1p and *Wd*yg1p, where the surface‐exposed catalytic center is subject to saponification of the acyl‐enzyme complex (Figure 5a; Figure S1, Supporting Information), the transition to the closed‐state might enable an intramolecular Claisen cyclization/Dieckmann condensation reaction in *Pl*AntI^closed^. Here, the polar network acts as a base to deprotonate C2′. Rearrangement to the enolate tautomer facilitates the cyclization reaction (C2′, (**II**)). The negative charge of the O1′ might be stabilized by the backbone of the GLDS loop and the sidechain of Ser176. Next, the C2' atom of the enolate group attacks C2″ in the acyl‐enzyme complex (**III**), and the tetrahedral coordination of the intermediate with the oxyanion hole (Leu174NH and Phe246NH) triggers the Dieckmann condensation (**IV**). Upon completion of the intramolecular cyclization, the covalent bond between ligand and enzyme is broken via proton transfer from His355 to Ser245. At the same time, the repulsion between the anthrone and enzyme converts *Pl*AntI^closed^ back to the open conformation. Eventually, both products leave the chamber, and the cyclic heptaketide oxidizes and aromatizes spontaneously to AQ‐256, which can no longer bind to *Pl*AntI^open^. This mechanistic model, illuminated by this study's detailed structural insights, refines the broad reaction trajectory inferred from previous modeled reaction intermediates and quantum chemical density functional theory calculations.^[^ ^5^ ^]^

Taken together, our results reveal atomic details of the biosynthesis of key metabolites during AQ and DHN‐melanin formation in bacteria and fungi. A common step during their assembly is polyketide trimming, which Afyg1p, Wdyg1p, and PlAntI catalyze. Despite numerous similarities in this class of enzymes, minor changes such as gating, ACP release, or subsequent reactions lead to entirely different reaction sequences. Thus, our study provides remarkable insights into how nature uses a simple toolbox to produce various polyaromatic building blocks for pigment syntheses in prokaryotes and eukaryotes.

## Conflict of Interest

The authors declare no conflict of interest.

## Supporting information

Supporting Information

## Data Availability

The data that support the findings of this study are available from the corresponding author upon reasonable request.
