# Supplementary material for: Polyketide Trimming Shapes Dihydroxynaphthalene‐Melanin and Anthraquinone Pigments
Source: Adv Sci (Weinh). 2024 Mar 16;11(22):2400184. doi: 10.1002/advs.202400184 (PMC11165489; doi:10.1002/advs.202400184)
Supplement: Supplementary file 1 — Supporting Information [file ADVS-11-2400184-s001.pdf]

ADVANCED SCIENCE
Open Access

Supporting Information

for *Adv. Sci.*, DOI 10.1002/advs.202400184

Polyketide Trimming Shapes Dihydroxynaphthalene-Melanin and Anthraquinone Pigments

Maximilian Schmalhofer, Anna L. Vagstad, Qiuqin Zhou, Helge B. Bode and Michael Groll**

Supplemental Information

Polyketide trimming shapes dihydroxynaphthalene-melanin and anthraquinone pigments

Maximilian Schmalhofer[1]*, Anna L. Vagstad[2], Qiuqin Zhou[3,8], Helge B. Bode[3-7], and Michael Groll[1]*

Table of Contents

General Methods..2

Figure S1 Open states of *Af*yg1p and *Wd*yg1p and closed/open states of *Pl*AntI 4
Figure S2 *Pl*AntI mutations alter AQ-256 production 5
Figure S3 Purification and crystallization of enzymes...6
Figure S4 Sequence alignments of *Af*yg1p and *Wd*yg1p with *Pl*AntI 7
Figure S5 *Af*yg1p and *Wd*yg1p are involved in the biosynthesis of 1,8-DHN melanin.........8

Table S1 Molecular cloning 9
Table S2 Primers used in this study 10
Table S3 Expression plasmids used in this study...10
Table S4 Buffers used in this study 10
Table S5 Minimal medium and additives 11
Table S6 Crystallization conditions...10
Table S7 Compounds used for protein:ligand complexes...11
Table S8 Crystallization conditions of protein:ligand complexes.....................................12
Table S9 Crystallographic data collection and refinement statistics................................13
Table S10 Crystallographic data collection and refinement statistics................................14
Table S11 Crystallographic data collection and refinement statistics................................15
Table S12 Crystallographic data collection and refinement statistics................................16

References..17

1. General methods

Chemicals

Unless stated otherwise, all chemicals were purchased from Sigma-Aldrich (St. Louis, US), AppliChem (Darmstadt, DE), Serva (Heidelberg, DE), Carl Roth (Karlsruhe, DE), Merck (Darmstadt, DE), or VWR (Radnor, US).

Cloning and mutagenesis

Eurofins Genomics synthesized the codon-optimized gene sequences for *Af*yg1p and *Wd*yg1p (Ebersberg, Germany, **Tabel S1**). They were inserted into expression vectors using restriction enzyme-based cloning (Primers are listed in **Table S2**). The generated plasmids (pRSET A^{AMP}-TEV-His_6-*Af*yg1p and $pCDF^{Spec}$-TEV-Strep-*Wd*yg1p, **Table S3**) were confirmed by Sanger-sequencing (GATC, Eurofins Genomics, Ebersberg, Germany) and transformed into *E. coli* BL21gold(DE3) by electroporation. Mutagenesis was performed using the QuikChange Site-

Directed Mutagenesis Kit according to the manufacturer's instructions (Primers are listed in **Table S2**). Generation and Mutagenesis of the modified *E. coli* strains were described by Zhou et al.[1]

Recombinant expression and purification

His_6-TEV-*Af*yg1p and Strep-TEV-*Wd*yg1p were expressed overnight in BL21gold(DE3) cells at 20 °C using isopropyl-β-D-1-thiogalactopyranoside (IPTG, 1 mM final concentration) for induction. The methionine feedback inhibition method was used to produce the selenomethionine-labeled protein.[2] In brief, the main culture was grown in minimal medium (media composition is listed in **Table S5**) instead of LB and at an OD_{600} of 0.6–0.8, the feedback inhibition amino acids mix (**Table S5**) and after another 15 minutes, IPTG was added. The cells were harvested and washed with an isotonic solution of sodium chloride. A 5 g cell pellet was thawed on ice and resuspended in 50 ml buffer A (respective buffers of all constructs are listed in **Table S4**). Without the addition of protease inhibitor, the cells were lysed on ice by sonication (5 minutes, 70 % amplitude, 1 second on/off and centrifuged at 40,000 g for 20 minutes at 4 °C. The supernatant was applied to an affinity chromatography column (HisTrap^{HP} or StrepTrap^{HP} 5 ml, Cytiva, Marlborough, US) that had been previously equilibrated with buffer A. The bound protein was washed with buffer A and eluted with the respective buffer B (**Table S4**). Next, *Af*yg1p and *Wd*yg1p were dialyzed at 4 °C overnight against buffer C and loaded onto a size-exclusion chromatography column (Superdex HiLoad 16/600 pg 75 or 200, Cytiva, Marlborough, US) that had been equilibrated with buffer C (**Table S4**). The combined fractions were concentrated to 30–50 mg/ml using a 30 kDa molecular weight cut-off (MWCO) Amicon Ultra-15 Centrifugal Filter and flash frozen in liquid nitrogen for storage at -80 °C. The protein quality was analyzed in all steps with SDS-PAGE (**Figure S3**). The purification of selenomethionine-labeled protein was performed identically to the native protein (see below).

Crystallization

The sitting drop vapor diffusion method was applied to screen for initial crystallization conditions at 20 °C. The 96-well Intelli-Plates (Art Robbins Instruments, Sunnyvale, US) with NeXtal Tubes Suite screens (Qiagen, Hilden, DE) were prepared with a Crystal Liquid Handling System Phoenix or Gryphon (Art Robbins Instruments, Sunnyvale, US). Hereafter, protein and reservoir solutions were mixed (0.2 µl + 0.2 µl, 0.2 µl + 0.1 µl, and 0.3 µl + 0.1 µl) with the Crystal Phoenix or Gryphon or the Oryx4 Protein Crystallization Robot (Douglas Instruments, East Garston, UK). Identified crystallization parameters were further optimized by applying the hanging drop vapor diffusion method. After adding ethylene glycol as a cryoprotectant (15-30 % (v/v)), the crystals were vitrified in liquid nitrogen and used to collect diffraction data at the SLS, Villigen, CH (beamline X06SA at Paul Scherrer Institute). The optimized crystallization conditions of all constructs are summarized in **Table S6**.

Incorporation of ligands

Crystals of all described protein:ligand complex structures were grown under similar conditions to native protein. The used ligands are listed in **Table S7**. The applied methods were soaking or co-crystallization experiments. The main parameters screened in hanging-drop vapor diffusion experiments were pH, solvent, ligand or protein concentration, and soaking duration. The soaking and co-crystallization conditions of all protein:ligand structures are summarized in **Table S8**. Notably, *Wd*yg1p predominantly crystallized as tiny needles that were hardly reproducible. However, in the presence of ligands (e.g., DHNs or THN), reproducible growth of crystals was accomplished even without the ligand resolved in the crystal structures.

Structure determination and data analysis

The diffraction images were recorded using synchrotron radiation (λ = 1.0 Å) at the beamline X06SA of the Swiss Light Source (SLS, Paul Scherrer Institute, Villigen, CH). The obtained

reflection intensities were evaluated with the XDS suite, and data reductions were performed using XSCALE.[3] If necessary, the topology and geometric restraints of the ligands were calculated using ProDRG or AceDRG.[4] Solvent content and the number of protomers were estimated based on the calculation of the Matthews coefficients with consideration of molecular weight and oligomeric state of the proteins and the crystal parameters (**Table S9** to **Table S12**).[5] Experimental phases for *Af*yg1p were determined by single-wavelength anomalous dispersion (SAD) methods at the peak absorption wavelength of selenium incorporated into a single crystal of *Af*yg1p (λ = 0.97914 Å, f' = -7.58, f'' = 5.56). CRANK2 was used for the automatic X-ray structure solution of the anomalous dataset (2.2 Å resolution, **Table S10**).[6] The hereby obtained model was sufficient for the phase determination of *Af*yg1p^{apo} and *Wd*yg1p^{apo}. The structures were optimized by restrained refinements in iterative rounds (REFMAC5 or PHENIX), and model building was carried out with Coot, Main, or PHENIX Autobuild.[7] Water molecules were automatically placed using ARP/wARP solvent.[8] The obtained X-ray structures were validated using the online tools MolProbity, PROCHECK, and wwPDB Validation System, and the statistics are summarized in **Tables S9** to **Table S12**.[9] Structural superpositions and rmsd values were calculated for the C^{α} backbone using Top3D.[10]

Figure illustration

Figures and structural representations were prepared with CorelDRAW 2019 Version 21.0.0.593 (Corel Corporation, Ottawa, CA) and the PyMOL Molecular Graphics System Version 2.4.0 (Schrödinger, New York, US), respectively. Protein and active-site residues are shown as white and yellow sticks, respectively. Ligands are illustrated in sticks and colored pink (1-N), yellow (1,3-DHN), green (1,3,6,8-THN), purple (PMSF), light green (DHA), and dark green (Ac), respectively. H-bonding and pi-stacking interactions are indicated as black dots and circle lines. Electron density maps are represented as $2F_o$-F_c (blue mesh, contoured to 1 σ), positive (green mesh, contoured to 3 σ) or negative (red mesh, contoured to -3 σ) F_o-F_c difference maps with respective ligands or residues omitted prior to phasing. Multiple sequence alignment was performed and illustrated by the Clustal Omega and ESPript servers, respectively.[11]

Figure S1. Open states of *Af*yg1p and *Wd*yg1p and closed/open states of *Pl*AntI. Shown are the surface representations of *Pl*AntI:1N (open) and *Pl*AntI^{closed}, *Af*yg1p^{PMSF}, *Af*yg1p^{apo}, *Af*yg1p:1,3-DHN, *Af*yg1p:THN, *Wd*yg1p^{apo}, *Wd*yg1p:1,3-DHN, and *Wd*yg1p:THN (from top left to bottom right). The closed state of *Pl*AntI^{closed} is shown in a box.

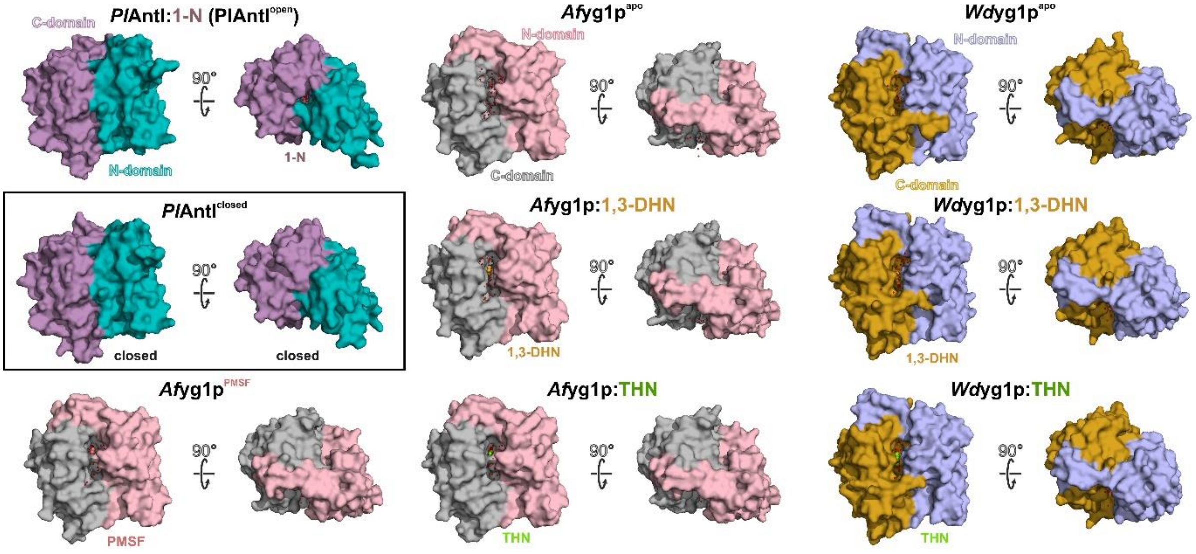

Figure S2. *Pl*AntI mutations alter AQ-256 production. a) HPLC/MS analyses and extracted ion chromatograms (EICs, AQ-256, solid line, *m/z* 255 [M-H]$^-$ and aloesaponarin II, SP^{EC}2, dotted line, *m/z* 253 [M-H]$^-$). of *E. coli* expressing *ant* BGC (*ant*ABCDEFGH) with the AntI mutants Ser245Ala, Asp326Ala, His355Ala, and the gating deficient mutant Asp327Ala, respectively. Figure is extended from Zhou et al.[5] **b)** Chemical reaction forming the shunt metabolite 3,8-dihydroxy-1-methyl-anthraquinone-2-carboxylic acid (DMAC/SP^{EC}1) and the decarboxylated derivative aloesaponarin II (SP^{EC}2).[5, 12]

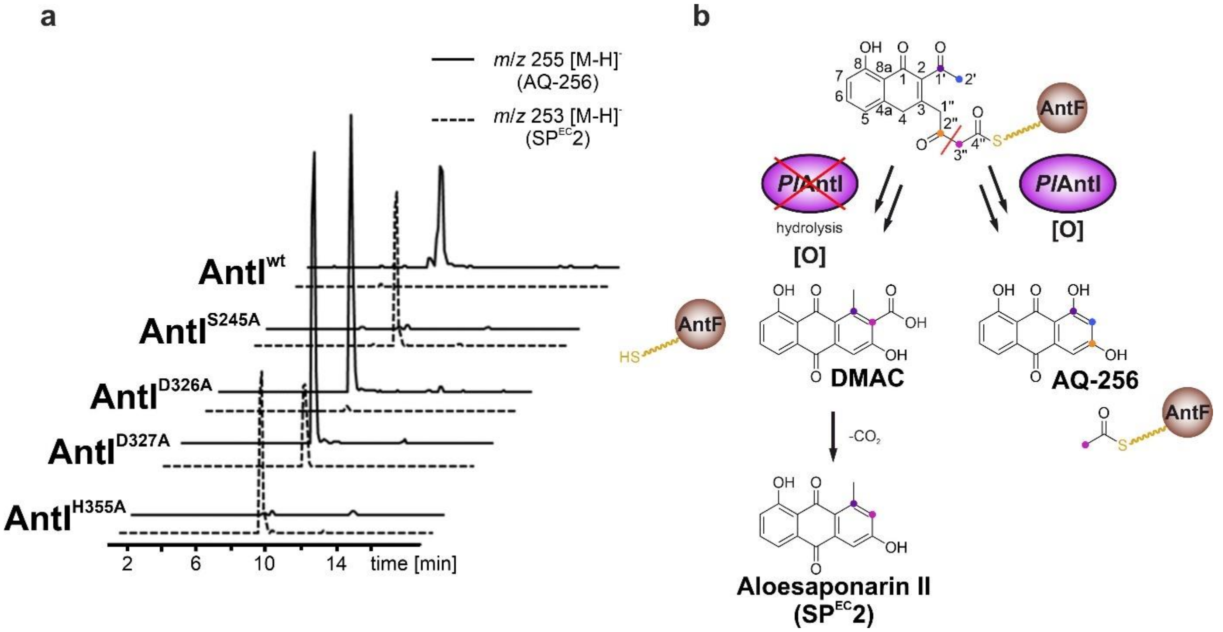

Figure S3. Purification and crystallization of enzymes. a) Size-exclusion chromatography (SEC) and sodium dodecyl sulfate polyacrylamide gel electrophoresis (SDS-PAGE) of *Af*yg1p (black line), *Wd*yg1p (long, dashed line), and *Pl*AntI (short, dashed line). **b)** Crystals in *apo* (left panel), in presence of 1,3-dihydroxynaphthalene (1,3-DHN, middle panel) or 1,3,6,8-tetrahydroxynaphthalene (THN, right panel).

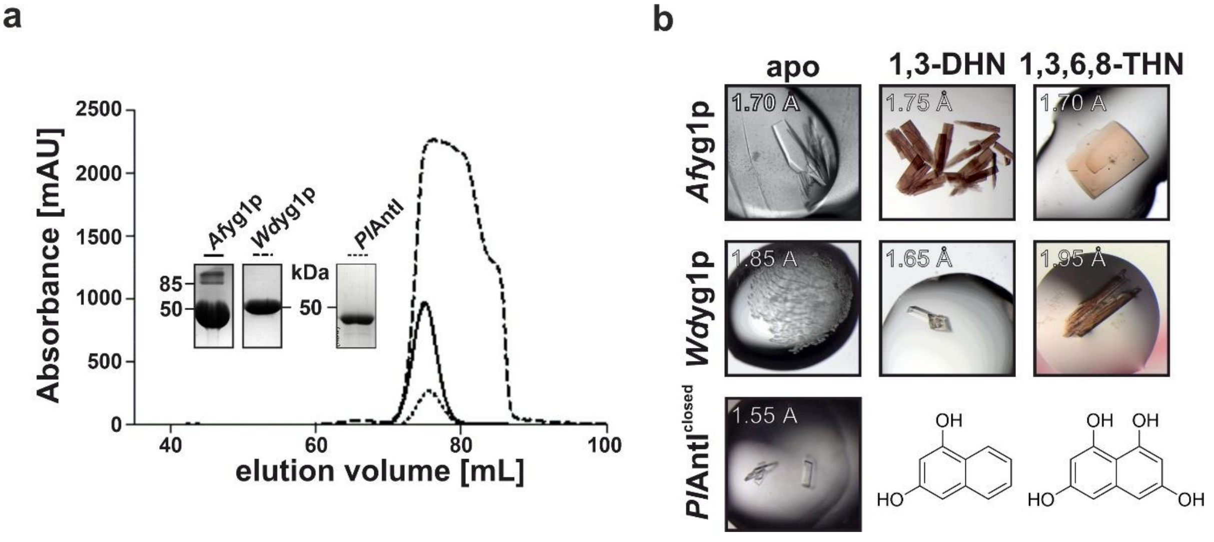

Figure S4. Sequence alignments of *Af*yg1p and *Wd*yg1p with *Pl*AntI. Red arrows, black squares, and black triangles emphasize the active-site residues, the GXSXG esterase (S is the

active-site nucleophile), and the GLD(G/S) β-turn motif, respectively. The alignment includes the amino acid sequences of *Af*yg1p (AAF03354.1, *Aspergillus fumigatus*), *Wd*yg1p (XP_009154598.1, *Exophiala* (*Wangiella*) *dermatitidis* NIH/UT8656), and *Pl*AntI (WP_011148290.1, *Photorhabdus luminescens* subsp. *laumondii* TT01).

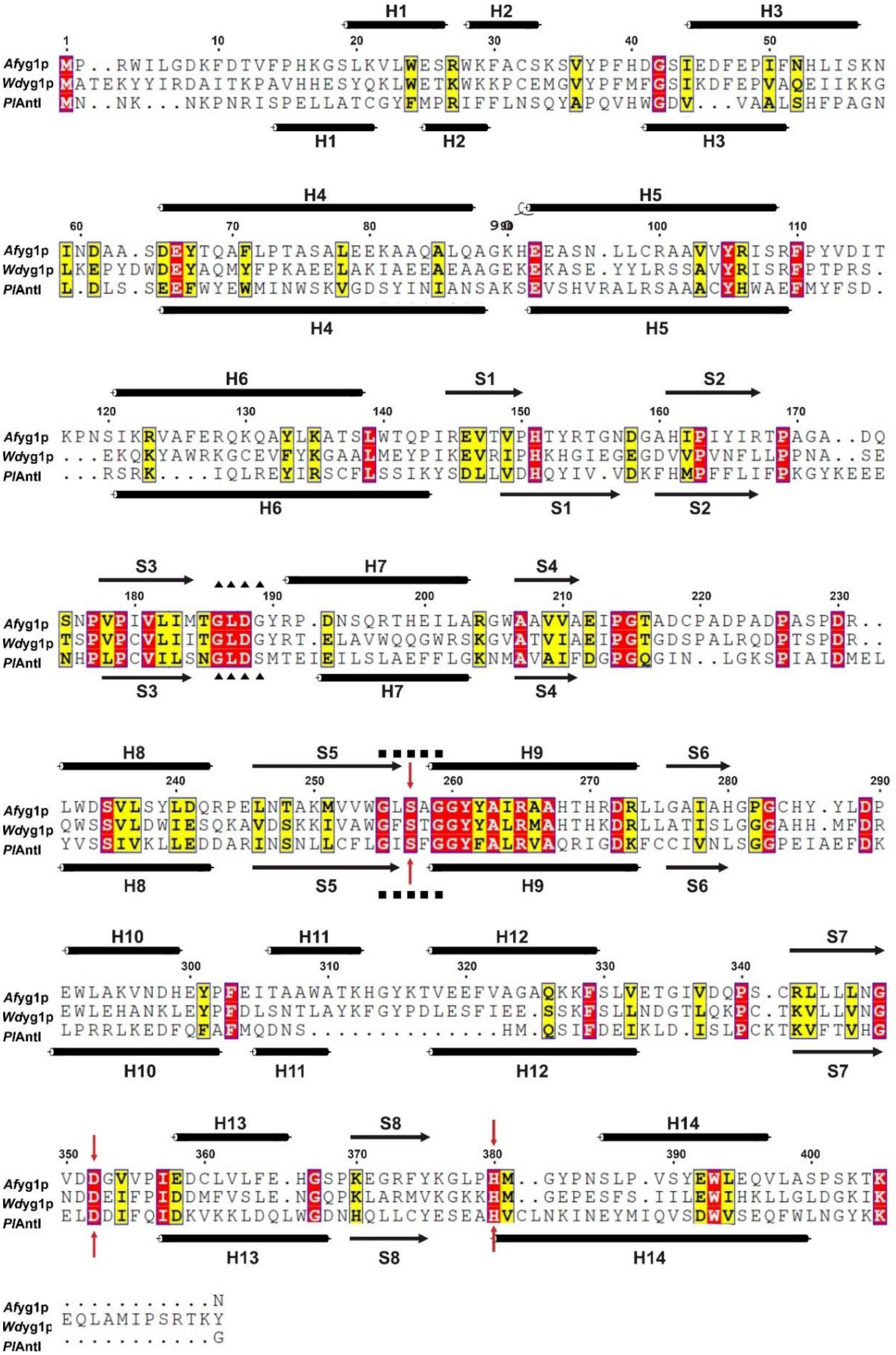

Figure S5. *Af*yg1p and *Wd*yg1p are involved in the biosynthesis of 1,8-DHN melanin. a) Structural superposition of *Af*yg1p in complex with PMSF, 1,3-DHN, and THN depicted as red,

orange, and green sticks, respectively. Water molecules are shown as spheres and colored according to the respective ligands. Tyr383 rotates by 90° in the presence of dehydroxylated surrogates in the *Af*yg1p:1,3-DHN complex structure and is highlighted in gray. **b)** Stick representation of the active site in *Af*yg1p^{apo}. Water molecules are illustrated as red spheres. **c)** The active site of *Af*yg1p^{PMSF} (white sticks) superimposed onto *Wd*yg1p^{apo} (gray sticks) highlights differences in the oxyanion holes. H-bonding interactions of Thr257 in *Wd*yg1p are presented in green dots. Notably, the oxyanion hole in the wild-type enzymes is occupied by water molecules (*Wd*yg1p, gray sphere; *Af*yg1p, orange sphere).

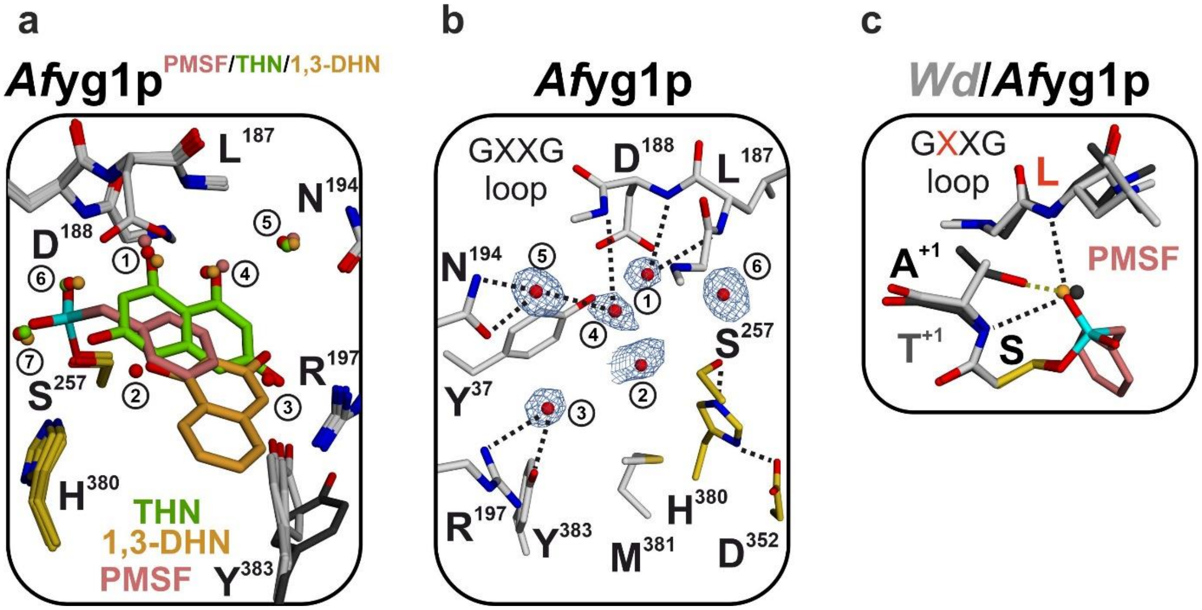

Figure S6. Mass spectrometry (MS) unveils the transformation of *Pl*AntI's active site serine to dehydroalanine (DHA) in the presence of phenylmethanesulphonyl fluoride (PMSF). a) The calculated molecular mass (m) of intact *Pl*AntI (excluding the start methionine) is 45841.4 g mol^{-1}. **b)** Following a one-hour incubation with PMSF, PlAntI exhibits an 18 Da reduction in mass (m = 45823.4 g mol^{-1}) compared to the wild type protein, indicating a condensation reaction with water splitting.

a

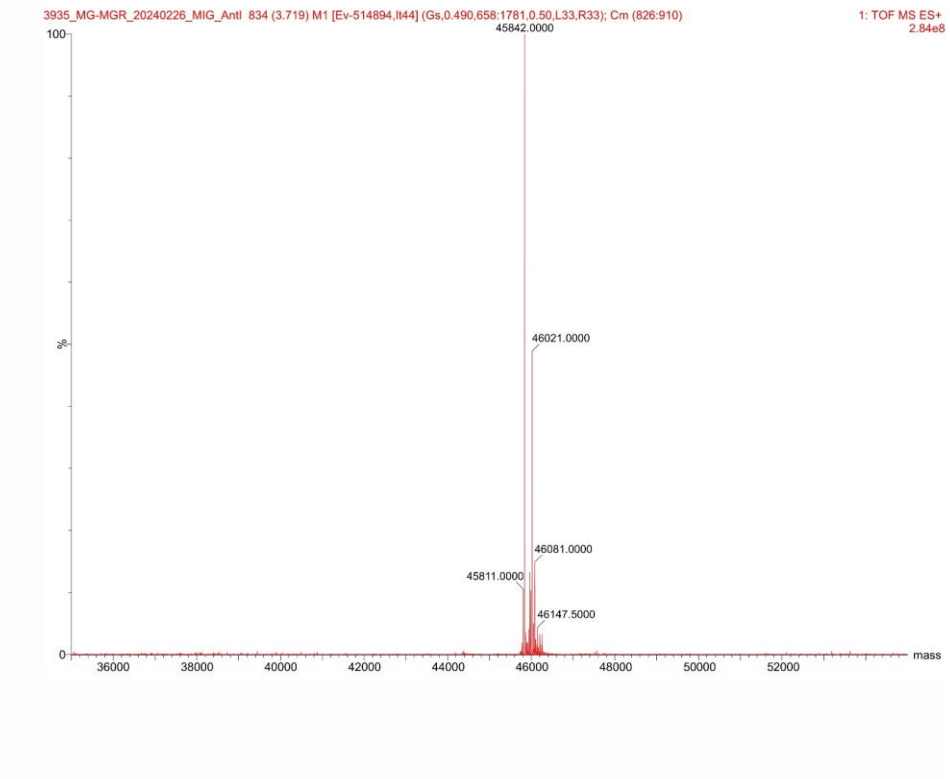

b

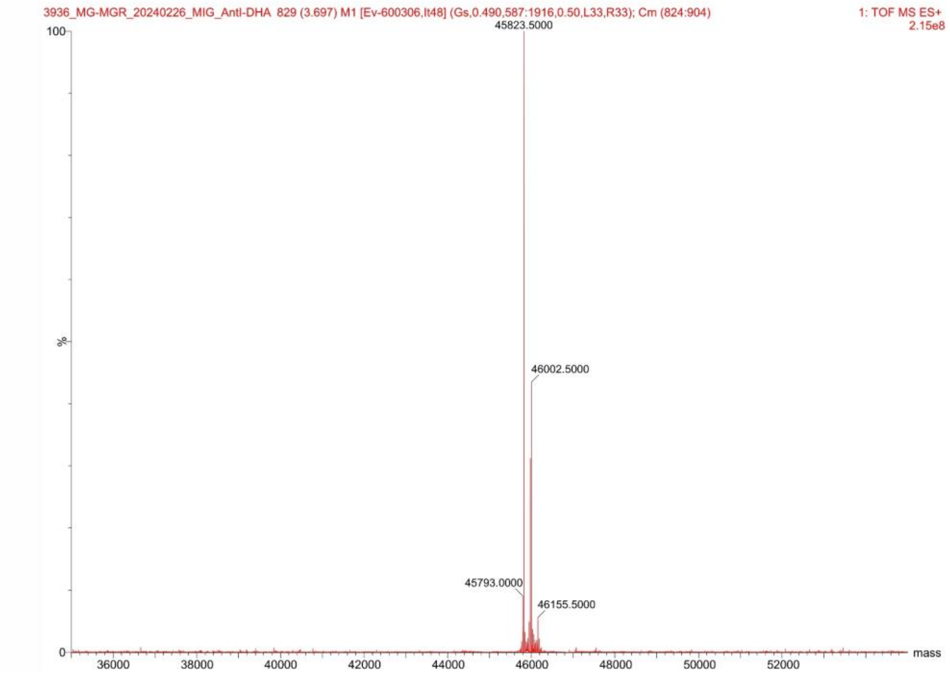

Table S1. Molecular cloning. Optimized genes used for molecular cloning of *Af*yg1p (*Aspergillus fumigatus*) and *Wd*yg1p (*Exophiala dermatitidis*) constructs.

***Af*yg1p (GenBank: AF116902.1)**
CCG CGT TGG ATC TTA GGT GAT AAA TTT GAC ACC GTA TTC CCG CAC AAA GGC TCT
CTG AAA GTT CTG TGG GAA TCT CGT TGG AAA TTC GCT TGC TCT AAA TCT GTT TAC
CCG TTC CAC GAC GGT TCT ATC GAA GAC TTC GAA CCG ATC TTC AAC CAC CTG ATC
TCT AAA AAC ATC AAC GAC GCT GCT TCT GAC GAA TAC ACC CAG GCT TTC CTG CCG
ACC GCT TCT GCT CTG GAA GAA AAA GCT GCT CAG GCT CTG CAA GCT GGT AAA CAC
GAA GAA GCT AGT AAC CTG CTG TGC CGT GCT GCT GTT GTT TAC CGT ATC TCT CGT
TTC CCG TAC GTT GAC ATC ACC AAA CCG AAC TCT ATC AAA CGT GTT GCT TTC GAA
CGT CAG AAA CAG GCT TAC CTG AAA GCT ACC TCT CTG TGG ACC CAG CCG ATC CGT
GAA GTT ACC GTT CCG CAC ACC TAC CGT ACC GGT AAC GAC GGT GCT CAC ATC CCG
ATC TAC ATC CGT ACC CCG GCT GGT GCT GAC CAG TCT AAC CCG GTT CCG ATC GTT
CTG ATC ATG ACC GGT CTG GAC GGT TAC CGT CCG GAC AAC TCT CAG CGT ACC CAC
GAA ATC CTG GCT CGT GGT TGG GCT GCT GTT GTT GCT GAA ATC CCG GGT ACC GCT
GAC TGC CCG GCT GAC CCG GCT GAC CCG GCT TCT CCG GAC CGT CTG TGG GAC TCT
GTT CTG TCT TAC CTG GAC CAG CGT CCG GAA CTG AAC ACC GCT AAA ATG GTT GTT
TGG GGT CTG TCT GCT GGT GGT TAC TAC GCT ATC CGT GCT GCT CAC ACC CAC CGT
GAC CGT CTG CTG GGT GCT ATC GCT CAC GGT CCG GGT TGC CAC TAC TAC CTG GAC
CCG GAA TGG CTG GCT AAA GTT AAC GAC CAC GAA TAC CCG TTC GAA ATC ACC GCT
GCT TGG GCT ACC AAA CAC GGT TAC AAA ACC GTT GAA GAA TTC GTT GCT GGT GCT
CAG AAA AAA TTC TCT CTG GTT GAA ACC GGT ATC GTT GAC CAG CCG TCT TGC CGT
CTG CTG CTG CTG AAC GGT GTT GAC GAC GGT GTT GTT CCG ATC GAA GAC TGC CTG
GTT CTG TTC GAA CAC GGT TCT CCG AAA GAA GGT CGT TTC TAC AAA GGT CTG CCG
CAC ATG GGT TAC CCG AAC TCT CTG CCG GTT TCT TAC GAA TGG CTG GAA CAG GTT
CTG GCT TCT CCG TCT AAA ACC AAA AAC TAA
***Wd*yg1p (Gene ID: 20306951)**
GCT ACC GAA AAA TAC TAC ATC CGT GAC GCT ATC ACC AAA CCG GCT GTT CAC CAC
GAA TCT TAC CAG AAA CTG TGG GAA ACC AAA TGG AAA AAA CCG TGC GAA ATG GGT
GTT TAC CCG TTC ATG TTC GGT TCT ATC AAA GAC TTC GAA CCG GTT GCT CAG GAA
ATC ATC AAA AAA GGT CTG AAA GAA CCG TAC GAC TGG GAC GAA TAC GCT CAG ATG
TAC TTC CCG AAA GCT GAA GAA CTG GCT AAA ATC GCT GAA GAA GCT GAA GCT GCT
GGT GAA AAA GAA AAA GCT AGT GAA TAC TAC CTG CGT TCT TCT GCT GTT TAC CGT
ATC TCT CGT TTC CCG ACC CCG CGT TCT GAA AAA CAG AAA TAC GCT TGG CGT AAA
GGC TGC GAA GTC TTC TAC AAA GGG GCT GCT CTG ATG GAA TAC CCG ATC AAA GAA
GTT CGT ATC CCG CAC AAA CAC GGT ATC GAA GGT GAA GGT GAC GTT GTT CCG GTT
AAC TTC CTG CTG CCG CCG AAC GCT TCT GAA ACC TCT CCG GTT CCG TGC GTT CTG
ATC ATC ACC GGT CTG GAC GGT TAC AGG ACC GAG CTG GCT GTG TGG CAG CAG GGG
TGG CGT TCT AAA GGT GTT GCT ACC GTT ATC GCT GAA ATC CCG GGT ACC GGT GAC
TCT CCG GCT CTG CGT CAG GAC CCG ACC TCT CCG GAC CGT CAG TGG TCT TCT GTT
CTG GAC TGG ATC GAA TCT CAG AAA GCT GTT GAC TCT AAA AAA ATC GTT GCT TGG
GGT TTC TCT ACC GGT GGT TAC TAC GCT CTG CGT ATG GCT CAC ACC CAC AAA GAC
CGT CTG CTG GCT ACC ATC TCT CTG GGT GGT GGT GCT CAC CAC ATG TTC GAC CGT
GAA TGG CTG GAA CAC GCT AAC AAA CTG GAA TAC CCG TTC GAC CTG TCT AAC ACC
CTG GCT TAC AAA TTC GGT TAC CCG GAC CTC GAA TCG TTC ATC GAA GAA TCG TCT
AAA TTC TCT CTG CTG AAC GAC GGT ACC CTG CAA AAA CCG TGC ACC AAA GTT CTG
CTG GTT AAC GGT AAC GAC GAC GAA ATC TTC CCG ATC GAC GAC ATG TTC GTT TCT
CTG GAA AAC GGT CAG CCG AAA CTG GCT CGT ATG GTT AAA GGT AAA AAA CAC ATG
GGT GAA CCG GAA TCT TTC TCT ATC ATC CTG GAA TGG ATA CAC AAA CTG CTG GGT
CTG GAC GGT AAA ATC AAA GAA CAG CTG GCT ATG ATC CCG TCT CGT ACC AAA TAA

Table S2. **Primers used in this study.**

**Sequencing Primer**

T7-terminator-R	`GCT AGT TAT TGC TCA GC GG`
pRSET RP-R	`ATG CTA GTT ATT GCT CAG C`
ACYCDuetUP1-F	`GGA TCT CGA CGC TCT CCC T`
DuetUP2-F	`TTG TAC ACG GCC GCA TAA TC`
DuetDOWN1-R	`GAT TAT GCG GCC GTG TAC AA`
**Molecular Cloning Primer**	
*Af*yg1p-BamHI-F	**CCA GGA TCC** CCG CGT TGG ATC TTA GGT G
*Af*yg1p-PstI-R	**CCA CTG CAG TTA** TTA GTT TTT GGT TTT AGA CGG AG
*Wd*yg1p-BamHI-F	**CCA GGA TCC** GCT ACC GAA AAA TAC TAC ATC CG
*Wd*yg1p-PstI-R	**CCA CTG CAG TTA** TTA TTT GGT ACG AGA CGG GAT C

Table S3. **Expression plasmids used in this study.**

**Expression vectors**	
pRSET A^{AMP}-TEV-His$_6$-*Af*yg1p	This work
pCDF^{Spec}-TEV-Strep-*Wd*yg1p	This work
ZQ40 pCOLA^{Kan}-TEV-His$_6$-*Pl*AntI	[6]

Table S4. **Buffers used in this study.**

**Buffers A and B for affinity chromatography**	
Buffer A$^{Wd\text{yg1p}}$	100 mM tris, 500 mM NaCl, pH 8.0, 5 mM DTT
Buffer A$^{Af\text{yg1p}}$	100 mM tris, 500 mM NaCl, pH 7.5, 20 mM imidazole, 5 mM □-ME
Buffer A$^{Pl\text{AntI}}$	100 mM tris, 300 mM NaCl, pH 8.0, 20 mM imidazole, 10 % (v/v) glycerol, 2 mM □-ME
Buffers B$^{Wd\text{yg1p}}$	Same as buffer A$^{Wd\text{yg1p}}$ + 2.5 mM *d*-Desthiobiotin
Buffer B$^{Af\text{yg1p}}$	Same as buffer A$^{Af\text{yg1p}}$ + 200 mM imidazole
Buffer B$^{Pl\text{AntI}}$	Same as buffer B$^{Pl\text{AntI}}$ + 500 mM imidazole
**Buffers C for size-exclusion chromatography**	
Buffer C$^{Wd\text{yg1p}}$	20 mM tris, 100 mM NaCl, pH 8.0, 2 mM DTT
Buffer C$^{Af\text{yg1p}/Pl\text{AntI}}$	20 mM tris, 100 mM NaCl, pH 7.5, 2 mM DTT

Table S5. **Minimal medium and additives.** Recombinant production of selenomethionine-labeled protein.

**M9 minimal medium**	$Na_2HPO_4 \cdot 2\ H_2O$	42 mM
	KH_2PO_4	22 mM
	NaCl	8.5 mM
	NH_4Cl	9.3 mM
	$MgSO_4 \cdot 7\ H_2O$	2 mM
	Glucose	0.4 % (w/v)
	Vitamins (1,000×)	0.1 % (v/v)
	Trace elements (100×)	1 % (v/v)
**1L vitamins pH 7.0 (1000×)**	EDTA	5 g
	$FeCl_3$	0.8 g
	$ZnCl_3$	0.05 g

	$CuCl_3$	0.01 g
	$CoCl_2$	0.01 g
	H_3BO_3	0.01 g
	$MnCl_2$	1.6 g
	Ni_2SO_4	some
	Molybdic acid	some
**1L trace elements (100×)**	Riboflavin	1 g
	Niacinamide	1 g
	Pyridoxine monohydrate	1 g
	Thiamine	1 g
**Feedback inhibition (Amino acid mix for 3L)**	Lysine, threonine, phenylalanine	0.3 g
	Leucine, isoleucine, valine	0.15 g
	L(+) selenomethionine	0.15 g

Table S6. Crystallization conditions.

**Construct**	**c (mg/mL)**	**reservoir solution**	**ratio[a]**
*Wd*yg1p	20	0.1 M tris, pH 8.0–9.0, 22–25 % (w/v) PEG3350	1:1
*Af*yg1p^{SeMET}	31.5	0.1 M bistris, pH 5.5, 25 % (w/v) PEG3350	2:1
*Af*yg1p	15	0.1–0.2 M K/Na tartrate, 15–26 % (w/v) PEG3350	1:1
*Pl*AntI	20	0.1 M hepes, pH 7.0–8.0, 0.1 M NaOAc, pH 6.6	1:1

[a]Ratio of protein to reservoir solution

Table S7. Compounds used for protein:ligand complexes.

**Ligands**	
1,3,6,8-tetrahydroxynaphthalene (THN)	ChemScene, Monmouth Junction, US
Phenylmethane sulfonyl fluoride (PMSF)	AppliChem, Darmstadt, DE
Phenyl sulfonyl fluoride (PSF)	Sigma-Aldrich, St. Louis, US
1,3-dihydroxynaphthalene (1,3-DHN)	Sigma-Aldrich, St. Louis, US
1-naphthol (1-N)	Sigma-Aldrich, St. Louis, US

Table S8. Crystallization conditions of protein:ligand complexes.

**Protein:ligand**	**c [mg/mL] (protein)**	**reservoir solution**	**c^{final} [mM] (ligand)**	**V^{final} [µL] (ligand)**	**C^{stock} [mM] (ligand)**
Soaking:					
*Af*yg1p^{PMS}	25	0.15 M K/Na tartrate<br>25 % (w/v) PEG3350	4	2.5	7[a]
*Af*yg1p:1,3-DHN	50	0.1 M K/Na tartrate<br>25 % (w/v) PEG3350	10	0.1	200
*Wd*yg1p^{DHA,*}	31	0.1 M tris, pH 9.2<br>22 % (w/v) PEG3350	4	2.5	7[a,c]
*Wd*yg1p$^{DHA,\#}$	29	0.1 M tris, pH 9.0<br>23 % (w/v) PEG3350	5	0.3	33.3[b,d]
*Pl*AntI^{DHA,*}	35	0.1 M hepes, pH 7.1<br>0.1 M NaOAc, pH 6.6<br>24 % (w/v) PEG3350	5	0.1	100

*Pl*AntI$^{\text{DHA,\#}}$	25	0.1 M hepes, pH 7.1<br>0.1 M NaOAc, pH 6.6<br>24 % (w/v) PEG3350	5	0.1	100
*Pl*AntI:1-N	15	0.1 M hepes, pH 7.9<br>0.1 M NaOAc, pH 6.6<br>21 % (w/v) PEG3350	10	0.1	200
Co-crystallization					
*Af*yg1p:THN	20	0.1 M K/Na tartrate<br>25.5 % (w/v) PEG3350	1		
*Wd*yg1p:1,3-DHN	20	0.1 M tris, pH 8.7<br>24 % (w/v) PEG3350	0.5		
*Wd*yg1p:THN	20	0.1 M tris, pH 8.5<br>21 % (w/v) PEG3350	0.5		

Used sulfonyl fluorides: PMSF (*) and PSF (#). Stock solutions of ligands (100 mM) were diluted with reservoir 1:14 (a) and 1:3 (b). Soaking experiments were performed overnight at 20 °C. Exceptions were [c] and [d] with 2 and 6 hours, respectively.

Table S9. **Crystallographic data collection and refinement statistics.**

	***Pl*AntI:1-N**	***Pl*AntI$^{\text{DHA,*}}$**	***Pl*AntI$^{\text{DHA,\#}}$**	***Pl*AntI^{closed}**
**Crystal parameters**				
Space group	$C222_1$	$C222_1$	$C222_1$	$C222_1$
Cell constants	a = 55.9 Å<br>b = 154.7 Å<br>c = 92.1 Å;	a = 54.7 Å<br>b = 155.0 Å<br>c = 91.4 Å;	a = 54.6 Å<br>b = 154.6 Å<br>c = 91.5 Å;	a = 54.5 Å<br>b = 154.7 Å<br>c = 91.4 Å;
Subunits / AU[a]	1	1	1	1
**Data collection**				
Beamline	X06SA, SLS	X06SA, SLS	X06SA, SLS	X06SA, SLS
Wavelength (Å)	1.0	1.0	1.0	1.0
Resolution range (Å)[b]	30-2.05<br>(2.15-2.05)	30-1.7<br>(1.8-1.7)	30-1.8<br>(1.9-1.8)	30-1.55<br>(1.65-1.55)
No. observations	110560	199233	168300	2500028
No. unique reflections[c]	24440	42427	35748	55924
Completeness (%)[b]	95.8 (96.8)	98.3 (98.5)	98.4 (99.4)	99.1 (99.9)
R_{merge} (%)[b, d]	4.5 (66.9)	4.8 (62.4)	4.6 (64.3)	2.9 (56.5)
I/σ (I)[b]	17.3 (3.8)	15.6 (2.5)	18.2 (2.3)	22.5 (2.4)
**Refinement (REFMAC5)**				
Resolution range (Å)	30-2.05	30-1.7	30-1.8	30-1.55
No. refl. working set	23215	40299	33957	53121
No. refl. test set	1221	2121	1787	2796
No. non-hydrogen atoms	3111	3252	3328	3394
No. of ligand atoms	11	5	5	-
No. of solvent atoms	98	246	317	355
R_{work}/R_{free} (%)[e]	19.1 / 20.7	16.6 / 18.8	16.1 / 19.5	16.1 / 17.9
r.m.s.d. bond (Å) / (°)[f]	0.002 / 1.1	0.003 / 1.1	0.003 / 1.1	0.003 / 1.1
Average B-factor (Å^2)				
Protein	56.3	35.8	34.3	30.5
Ligand	51.3	27.3	30.3	-
Solvent	52.2	41.8	43.1	40.6
Ramachandran Plot (%)[g]	97.6 / 2.4 / 0	98.1 / 1.9 / 0	98.4 / 1.6 / 0	97.9 / 2.1 / 0

PDB accession code	8QBH	8QD6	8QD5	8QBI

[a] Asymmetric unit

[b] The values in parentheses for resolution range, completeness, R_{merge}, and I/σ (I) correspond to the highest resolution shell

[c] Data reduction was carried out with XDS and from a single crystal. Friedel pairs were treated as identical reflections

[d] $R_{merge}(I) = \Sigma_{hkl} \Sigma_j |[I(hkl)_j - <I(hkl)>]| / \Sigma_{hkl} \Sigma_j I(hkl)_j$, where $I(hkl)_j$ is the jth measurement of the intensity of reflection hkl and <I(hkl)> is the average intensity

[e] $R = \Sigma_{hkl} | |F_{obs}| - |F_{calc}| | / \Sigma_{hkl} |F_{obs}|$, where R_{free} is calculated for a randomly chosen 5% of reflections, which were not used for structure refinement, and R_{work} is calculated for the remaining reflections

[f] Deviations from ideal bond lengths/angles

[g] Percentage of residues in favored/allowed/outlier region

The sulfonyl fluorides used were PMSF (*) and PSF ($^{\#}$).

Table S10. **Crystallographic data collection and refinement statistics.**

	*Af*yg1p^{SeMet}	*Af*yg1p^{apo}	*Wd*yg1p^{apo}
**Crystal parameters**			
Space group	$P2_12_12_1$	$P2_1$	$P2_12_12_1$
Cell constants	a = 85.3 Å	a = 85.8 Å	a = 77.0 Å
	b = 92.1 Å	b = 108.5 Å	b = 86.6 Å
	c = 93.3 Å	c = 91.9 Å	c = 115.7 Å
		β = 90.03°	
Subunits / AU[a]	2	4	2
**Data collection**			
Beamline	X06SA, SLS	X06SA, SLS	X06SA, SLS
Wavelength (Å)	0.97914	1.0	1.0
Resolution range (Å)[b]	30-2.2 (2.3-2.2)	30-1.7 (1.8-1.7)	30-1.85 (1.95-1.85)
No. observations	503398[†]	556406	295307
No. unique reflections[c]	71952	179315	65754
Completeness (%)[b]	99.9 (99.9)	97.2 (97.9)	98.6 (98.4)
R_{merge} (%)[b, d]	11.4 (54.6)	6.5 (52.4)	6.0 (54.3)
I/σ (I)[b]	11.6 (4.9)	9.6 (2.4)	16.1 (3.2)
**Refinement (REFMAC5)**			
Resolution range (Å)		30-1.7	30-1.85
No. refl. working set		170348	62450
No. refl. test set		8937	3287
No. non-hydrogen atoms		14233	7183
No. of ligand atoms		-	-
No. of solvent atoms		1743	602
R_{work}/R_{free} (%)[e]		16.5 / 19.8	15.6 / 19.7
r.m.s.d. bond (Å) / (°)[f]		0.003 / 1.2	0.003 / 1.1
Average B-factor (Å^2)			
Protein		25.2	28.4
Ligand		-	-
Solvent		32.1	34.3
Ramachandran Plot (%)[g]		96.5 / 3.5 / 0	96.2 / 3.8 / 0
PDB accession code		8QD1	8QD7

[a] Asymmetric unit

[b] The values in parentheses for resolution range, completeness, R_{merge}, and I/σ (I) correspond to the highest resolution shell

[c] Data reduction was carried out with XDS and from a single crystal. Friedel pairs were treated as identical reflections

[d] $R_{merge}(I) = \Sigma_{hkl} \Sigma_j |[I(hkl)_j - \langle I(hkl) \rangle]| / \Sigma_{hkl} \Sigma_j I(hkl)_j$, where $I(hkl)_j$ is the jth measurement of the intensity of reflection hkl and <I(hkl)> is the average intensity

[e] $R = \Sigma_{hkl} | |F_{obs}| - |F_{calc}| | / \Sigma_{hkl} |F_{obs}|$, where R_{free} is calculated for a randomly chosen 5% of reflections, which were not used for structure refinement, and R_{work} is calculated for the remaining reflections

[f] Deviations from ideal bond lengths/angles

[g] Percentage of residues in favored/allowed/outlier region

Table S11. Crystallographic data collection and refinement statistics.

	***Wd*yg1p:1,3-DHN**	***Wd*yg1p:THN**	***Wd*yg1p^{DHA,*}**	***Wd*yg1p$^{DHA,\#}$**
**Crystal parameters**				
Space group	$P2_12_12_1$	$P2_12_12_1$	$P2_12_12_1$	$P2_12_12_1$
Cell constants	a = 85.8 Å<br>b = 88.9 Å<br>c = 123.1 Å	a = 86.2 Å<br>b = 86.5 Å<br>c = 122.4 Å	a = 85.6 Å<br>b = 88.5 Å<br>c = 122.5 Å	a = 85.5 Å<br>b = 88.3 Å<br>c = 122.0 Å
Subunits / AU[a]	2	2	2	2
**Data collection**				
Beamline	X06SA, SLS	X06SA, SLS	X06SA, SLS	X06SA, SLS
Wavelength (Å)	1.0	1.0	1.0	1.0
Resolution range (Å)[b]	30-1.65<br>(1.75-1.65)	30-1.95<br>(2.05-1.95)	30-1.85<br>(1.95-1.85)	30-1.85<br>(1.95-1.85)
No. observations	493745	306939	301708	416769
No. unique reflections[c]	111963	67832	79470	78363
Completeness (%)[b]	98.7 (98.4)	98.5 (96.4)	99.3 (99.7)	98.6 (95.5)
R_{merge} (%)[b, d]	5.8 (65.2)	6.2 (63.8)	5.2 (53.8)	5.9 (63.1)
I/σ (I)[b]	11.8 (2.1)	14.1 (2.2)	15.9 (2.7)	16.0 (2.9)
**Refinement (REFMAC5)**				
Resolution range (Å)	30-1.65	30-1.95	30-1.85	30-1.85
No. refl. working set	106353	64426	75482	74434
No. refl. test set	5598	3391	3973	3918
No. non-hydrogen atoms	7431	7068	7279	7325
No. of ligand atoms	24	28	10	10
No. of solvent atoms	765	519	728	751
R_{work}/R_{free} (%)[e]	16.3 / 18.8	15.8 / 20.3	14.6 / 17.9	15.1 / 18.9
r.m.s.d. bond (Å) / (°)[f]	0.003 / 1.1	0.003 / 1.2	0.003 / 1.1	0.003 / 1.1
Average B-factor ($Å^2$)				
Protein	30.9	38.8	29.4	31.7
Ligand	37.4	45.1	28.6	28.9
Solvent	38.8	41.6	38.0	39.7
Ramachandran Plot (%)[g]	96.6 / 3.4 / 0	96.2 / 3.8 / 0	96.9 / 3.1 / 0	96.6 / 3.4 / 0
PDB accession code	**8QD8**	**8QD9**	**8QDA**	**8QDB**

[a] Asymmetric unit
[b] The values in parentheses for resolution range, completeness, R_{merge}, and I/σ (I) correspond to the highest resolution shell
[c] Data reduction was carried out with XDS and from a single crystal. Friedel pairs were treated as identical reflections
[d] $R_{merge}(I) = \Sigma_{hkl} \Sigma_j |[I(hkl)_j - \langle I(hkl)\rangle]| / \Sigma_{hkl} \Sigma_j I(hkl)_j$, where $I(hkl)_j$ is the jth measurement of the intensity of reflection hkl and <I(hkl)> is the average intensity
[e] $R = \Sigma_{hkl} | |F_{obs}| - |F_{calc}| | / \Sigma_{hkl} |F_{obs}|$, where R_{free} is calculated for a randomly chosen 5% of reflections, which were not used for structure refinement, and R_{work} is calculated for the remaining reflections
[f] Deviations from ideal bond lengths/angles
[g] Percentage of residues in favored/allowed/outlier region
The sulfonyl fluorides used were PMSF (*) and PSF (#).

Table S12. **Crystallographic data collection and refinement statistics.**

	***Af*yg1p:1,3-DHN**	***Af*yg1p:THN**	***Af*yg1p^{PMSF}**
**Crystal parameters**			
Space group	$P2_1$	$P2_1$	$P2_1$
Cell constants	a = 85.8 Å<br>b = 107.5 Å<br>c = 92.5 Å<br>β = 90.1°	a = 85.5 Å<br>b = 107.4 Å<br>c = 92.7 Å<br>β = 90.2°	a = 85.6 Å<br>b = 107.6 Å<br>c = 92.5 Å<br>β = 90.01°
Subunits / AU[a]	4	4	4
**Data collection**			
Beamline	X06SA, SLS	X06SA, SLS	X06SA, SLS
Wavelength (Å)	1.0	1.0	1.0
Resolution range (Å)[b]	30-1.75 (1.85-1.75)	30-1.7 (1.8-1.7)	30-1.8 (1.9-1.8)
No. observations	496678	556281	413528
No. unique reflections[c]	159406	173708	148528
Completeness (%)[b]	94.8 (96.2)	94.6 (95.7)	95.9 (97.4)
R_{merge} (%)[b, d]	10.9 (61.4)	9.4 (60.0)	6.6 (54.6)
I/σ (I)[b]	6.5 (2.1)	6.7 (1.9)	9.4 (1.9)
**Refinement (REFMAC5)**			
Resolution range (Å)	30-1.75	30-1.7	30-1.8
No. refl. working set	151446	164908	141275
No. refl. test set	7932	8775	7396
No. non-hydrogen atoms	13724	13719	12870
No. of ligand atoms	48	56	64
No. solvent atoms	1198	1176	434
R_{work}/R_{free} (%)[e]	22.9 / 23.8	22.5 / 25.0	27.8 / 29.2
r.m.s.d. bond (Å) / (°)[f]	0.003 / 1.2	0.002 / 1.2	0.003 / 1.1
Average B-factor ($Å^2$)			
Protein	22.3	22.2	29.3
Ligand	32.2	23.0	27.5
Solvent	28.6	28.3	27.8
Ramachandran Plot (%)[g]	96.5 / 3.5 / 0	96.2 / 3.8 / 0	95.9 / 4.1 / 0
PDB accession code	**8QD2**	**8QD3**	**8QD4**

[a] Asymmetric unit

[b] The values in parentheses for resolution range, completeness, R_{merge}, and I/σ (I) correspond to the highest resolution shell

[c] Data reduction was carried out with XDS and from a single crystal. Friedel pairs were treated as identical reflections

[d] $R_{merge}(I) = \Sigma_{hkl} \Sigma_j |[I(hkl)_j - <I(hkl)>]| / \Sigma_{hkl} \Sigma_j I(hkl)_j$, where $I(hkl)_j$ is the jth measurement of the intensity of reflection hkl and $<I(hkl)>$ is the average intensity

[e] $R = \Sigma_{hkl} | |F_{obs}| - |F_{calc}| | / \Sigma_{hkl} |F_{obs}|$, where R_{free} is calculated for a randomly chosen 5% of reflections, which were not used for structure refinement, and R_{work} is calculated for the remaining reflections

[f] Deviations from ideal bond lengths/angles

[g] Percentage of residues in favored/allowed/outlier region

References

[1] Q. Zhou, A. Bräuer, H. Adihou, M. Schmalhofer, P. Saura, G. L. C. Grammbitter, V. R. I. Kaila, M. Groll, H. B. Bode, *Chemical science* **2019**, *10*, 6341-6349.

[2] G. D. Van Duyne, R. F. Standaert, P. A. Karplus, S. L. Schreiber, J. Clardy, *Journal of molecular biology* **1993**, *229*, 105-124.

[3] a) W. Kabsch, *J. Appl. Cryst.* **1993**, *26*, 795-800; b) W. Kabsch, *Acta Crystallogr. Sect. D - Biol. Crystallogr.* **2010**, *66*, 125-132.

[4] a) A. W. Schüttelkopf, D. M. van Aalten, *Acta crystallographica. Section D, Biological crystallography* **2004**, *60*, 1355-1363; b) F. Long, R. A. Nicholls, P. Emsley, S. Graǽulis, A. Merkys, A. Vaitkus, G. N. Murshudov, *Acta crystallographica. Section D, Structural biology* **2017**, *73*, 112-122.
[5] B. W. Matthews, *Journal of molecular biology* **1968**, *33*, 491-497.
[6] a) W. A. Hendrickson, M. M. Teeter, *Nature* **1981**, *290*, 107-113; b) N. S. Pannu, W. J. Waterreus, P. Skubák, I. Sikharulidze, J. P. Abrahams, R. A. de Graaff, *Acta crystallographica. Section D, Biological crystallography* **2011**, *67*, 331-337; c) P. Skubák, N. S. Pannu, *Nature communications* **2013**, *4*, 2777.
[7] a) G. N. Murshudov, P. Skubák, A. A. Lebedev, N. S. Pannu, R. A. Steiner, R. A. Nicholls, M. D. Winn, F. Long, A. A. Vagin, *Acta crystallographica. Section D, Biological crystallography* **2011**, *67*, 355-367; b) P. D. Adams, P. V. Afonine, G. Bunkóczi, V. B. Chen, I. W. Davis, N. Echols, J. J. Headd, L. W. Hung, G. J. Kapral, R. W. Grosse-Kunstleve, A. J. McCoy, N. W. Moriarty, R. Oeffner, R. J. Read, D. C. Richardson, J. S. Richardson, T. C. Terwilliger, P. H. Zwart, *Acta crystallographica. Section D, Biological crystallography* **2010**, *66*, 213-221; c) D. Liebschner, P. V. Afonine, M. L. Baker, G. Bunkóczi, V. B. Chen, T. I. Croll, B. Hintze, L. W. Hung, S. Jain, A. J. McCoy, N. W. Moriarty, R. D. Oeffner, B. K. Poon, M. G. Prisant, R. J. Read, J. S. Richardson, D. C. Richardson, M. D. Sammito, O. V. Sobolev, D. H. Stockwell, T. C. Terwilliger, A. G. Urzhumtsev, L. L. Videau, C. J. Williams, P. D. Adams, *Acta crystallographica. Section D, Structural biology* **2019**, *75*, 861-877; d) P. Emsley, B. Lohkamp, W. G. Scott, K. Cowtan, *Acta Crystallogr. Sect. D - Biol. Crystallogr.* **2010**, *66*, 486-501; e) D. Turk, *Acta crystallographica. Section D, Biological crystallography* **2013**, *69*, 1342-1357.
[8] a) G. G. Langer, S. Hazledine, T. Wiegels, C. Carolan, V. S. Lamzin, *Acta crystallographica. Section D, Biological crystallography* **2013**, *69*, 635-641; b) V. S. Lamzin, K. S. Wilson, *Acta crystallographica. Section D, Biological crystallography* **1993**, *49*, 129-147.
[9] a) C. J. Williams, J. J. Headd, N. W. Moriarty, M. G. Prisant, L. L. Videau, L. N. Deis, V. Verma, D. A. Keedy, B. J. Hintze, V. B. Chen, S. Jain, S. M. Lewis, W. B. Arendall, 3rd, J. Snoeyink, P. D. Adams, S. C. Lovell, J. S. Richardson, D. C. Richardson, *Protein science : a publication of the Protein Society* **2018**, *27*, 293-315; b) R. A. Laskowski, M. W. MacArthur, D. S. Moss, J. M. Thornton, **1993**, *26*, 283-291; c) H. Berman, K. Henrick, H. Nakamura, *Nature structural biology* **2003**, *10*, 980.
[10] G. Lu, *Journal of Applied Crystallography* **2000**, *33*, 176-183.
[11] a) F. Sievers, A. Wilm, D. Dineen, T. J. Gibson, K. Karplus, W. Li, R. Lopez, H. McWilliam, M. Remmert, J. Söding, J. D. Thompson, D. G. Higgins, *Molecular systems biology* **2011**, *7*, 539; b) M. Goujon, H. McWilliam, W. Li, F. Valentin, S. Squizzato, J. Paern, R. Lopez, *Nucleic acids research* **2010**, *38*, W695-699; c) X. Robert, P. Gouet, *Nucleic acids research* **2014**, *42*, W320-324.
[12] M. Cummings, A. D. Peters, G. F. S. Whitehead, B. R. K. Menon, J. Micklefield, S. J. Webb, E. Takano, *PLoS biology* **2019**, *17*, e3000347.
